# The RPAP3-Cterminal domain identifies R2TP-like quaternary chaperones

**DOI:** 10.1038/s41467-018-04431-1

**Published:** 2018-05-29

**Authors:** Chloé Maurizy, Marc Quinternet, Yoann Abel, Céline Verheggen, Paulo E. Santo, Maxime Bourguet, Ana C.F. Paiva, Benoît Bragantini, Marie-Eve Chagot, Marie-Cécile Robert, Claire Abeza, Philippe Fabre, Philippe Fort, Franck Vandermoere, Pedro M.F. Sousa, Jean-Christophe Rain, Bruno Charpentier, Sarah Cianférani, Tiago M. Bandeiras, Bérengère Pradet-Balade, Xavier Manival, Edouard Bertrand

**Affiliations:** 1 IGMM, CNRS, Université de Montpellier, Montpellier, 34293 France; 2 Equipe labélisée Ligue Nationale Contre le Cancer, 34293, Montpellier, France; 3 CNRS, INSERM, IBSLOR, Université de Lorraine, Nancy, F-54000 France; 4 iBET, Instituto de Biologia Experimental e Tecnológica, Apartado 12, Oeiras, 2781-901 Portugal; 5 Instituto de Tecnologia Química e Biológica António Xavier, Universidade Nova de Lisboa, Av. da República, Oeiras, 2780-157 Portugal; 6 Laboratoire de Spectrométrie de Masse BioOrganique, Université de Strasbourg, CNRS, IPHC UMR 7178, Strasbourg, 67000 France; 7 CNRS, IMoPA, Université de Lorraine, Nancy, F-54000 France; 8 CRBM, University of Montpellier, CNRS, 1919 Route de Mende, Montpellier, 34090 France; 9 IGF, CNRS, Université de Montpellier, Montpellier, 34090 France; 10 Hybrigenics Services, Paris, 75014 France

## Abstract

R2TP is an HSP90 co-chaperone that assembles important macro-molecular machineries. It is composed of an RPAP3-PIH1D1 heterodimer, which binds the two essential AAA+ATPases RUVBL1/RUVBL2. Here, we resolve the structure of the conserved C-terminal domain of RPAP3, and we show that it directly binds RUVBL1/RUVBL2 hexamers. The human genome encodes two other proteins bearing RPAP3-C-terminal-like domains and three containing PIH-like domains. Systematic interaction analyses show that one RPAP3-like protein, SPAG1, binds PIH1D2 and RUVBL1/2 to form an R2TP-like complex termed R2SP. This co-chaperone is enriched in testis and among 68 of the potential clients identified, some are expressed in testis and others are ubiquitous. One substrate is liprin-α2, which organizes large signaling complexes. Remarkably, R2SP is required for liprin-α2 expression and for the assembly of liprin-α2 complexes, indicating that R2SP functions in quaternary protein folding. Effects are stronger at 32 °C, suggesting that R2SP could help compensating the lower temperate of testis.

## Abstract (web summary)

R2TP is an HSP90 co-chaperone composed of an RPAP3-PIH1D1 heterodimer, which binds two essential AAA+ ATPases RUVBL1/RUVBL2. Here authors use a structural approach to study RPAP3 and find an RPAP3-like protein (SPAG1) which also forms a co-chaperone complex with PIH1D2 and RUVBL1/2 enriched in testis.

## Introduction

The R2TP complex was discovered in *S. cerevisiae* as an HSP90 co-factor^1^. R2TP is an unusual co-chaperone because it appears specialized in quaternary protein folding, and in particular in the assembly of key cellular machineries important for cell growth^2^. Important R2TP clients are the small nucleolar ribonucleoprotein particles (snoRNPs), which are required for ribosomal RNAs maturation^3^. More recently, other substrates were described, including the U4 and U5 spliceosomal snRNAs^4–6^, the nuclear RNA polymerases^7–9^, and the family of PI3K-like kinases (PIKKs), which comprises mTOR, DNA-PK, ATR, ATM, SMG1 and TRRAP^10,11^. Given the role of these clients in cell growth and proliferation, it was hypothesized that R2TP mediates some of the tumorigenic effects elicited by HSP90^12^. Newly identified clients involved in DNA damage response corroborate this possibility^13^.

**Fig. 1:**
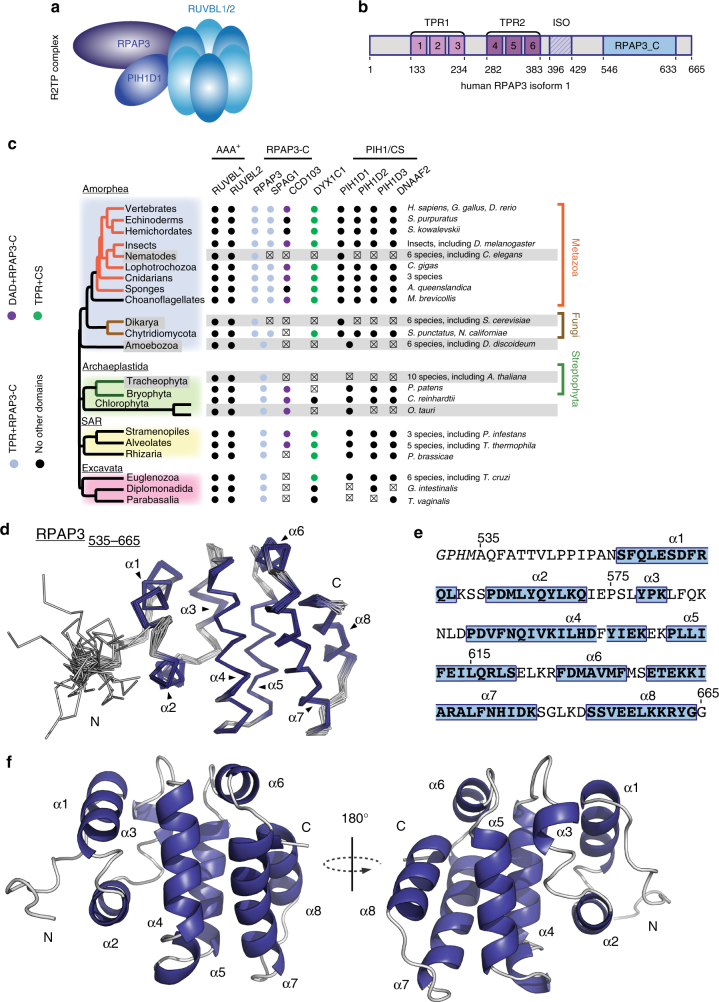
Solution structure of the C-terminal domain of human RPAP3 **a** Schematic representation of the human R2TP complex. **b** Domain architecture of RPAP3 (numbering corresponds to amino-acids of isoform 1). **c** Conservation of RPAP3 and PIH repertoires across Eukaryotes. Members in which TPR (Pfam: 13414) or Dynein attachment (Pfam: 15867) domains are associated to the canonical RPAP3_C are colored as indicated at the left. Clades or species that have lost flagella or in which cilia are not motile are in gray background. Members that were not found are indicated by x. **d** Backbone view of the superposition of the best 20 NMR structures of human RPAP3-Cter, with α-helices indicated in violet. **e** Sequence of RPAP3-Cter, with corresponding α-helices. **f** Backbone orthogonal views (180 °C) of RPAP3-Cter structure in ribbon representation with corresponding α-helices

In humans, R2TP is composed of a core that associates with prefoldins and additional factors to form the R2TP/Prefoldin-like complex, recently renamed PAQosome^2,7–9^. The R2TP core is composed of four subunits (Fig. 1a): PIH1D1, RPAP3, and the two related AAA+ATPases RUVBL1 and RUVBL2 (also called Pontin and Reptin). RPAP3 directly binds HSP90 and forms a stable heterodimer with PIH1D1^14,15^. These components are believed to function as adapter and regulatory factors, while RUVBL1 and RUVBL2 are catalytic components that likely possess a chaperone activity^16^. The analysis of snoRNP biogenesis suggests that R2TP functions through a stepwise process, where HSP90 stabilizes clients before their assembly, followed by the independent recruitment of several snoRNP components by R2TP, and ending in the loading of RUVBL1/2 on the client complex^17^. The molecular role of RUVBL1 and RUVBL2 still remains elusive. Structural studies showed that they form hexameric rings typical for this class of enzymes^18^. In addition, they contain an insertion called domain II, which protrudes from the ATPase ring and forms a flexible structure whose conformation depends on the presence and nature of bound nucleotides^19,20^. RUVBL1/2 make ATP-dependent contacts with some cofactors and client proteins^21,22^, suggesting that ATP loading and hydrolysis may act as a switch to control the binding and release of clients. Accordingly, it has been proposed that RUVBL1/2 stabilize interactions between subunits of target complexes^17^, or, alternatively, that they stimulate the dissociation of specific assembly factors^23^. Recent structural analyses showed that RUVBL1/2 cycle between hexameric and dodecameric forms, with client binding promoting dimerization of the hexamer, and ATP hydrolysis dissociation of the dodecamer^16^. This provides a glimpse of how these enzymes may chaperone their clients.

Structural studies revealed that PIH1D1 has two domains. Its N-terminal region harbors the conserved PIH domain that possesses a phospho-peptide binding pocket responsible for recognizing some substrates^14,15^. Its C-terminal region folds as a CS domain and mediates hetero-dimerization with RPAP3^14,15,24^. RPAP3 has a C-terminal domain of unknown function (RPAP3-Cter), and a middle region composed of six tetratricopeptide repeats (TPR) arranged in two consecutive domains (Fig. 1b). Structural studies showed that they bind HSP90 through five conserved residues forming a carboxylate clamp, which catches the last C-terminal residues of the chaperone (-MEEVD^15,25^). A recent cryo-EM structure of the yeast R2TP revealed that a single copy of the Tap1p:Pih1p dimer binds an hetero-hexamer of Rvb1/2 (the yeast homologs of RPAP3, PIH1D1 and RUVBL1/2, respectively^26,27^). This interaction involves the DII domain of the ATPases and the linker separating the PIH and CS domains of Pih1p^26–28^.

In this study, we elucidate the 3D structure of the RPAP3 C-terminal domain and we show that it binds directly the RUVBL1/2 ATPases. A similar domain is also present in a human protein called SPAG1, and we demonstrate that it forms an R2TP-like complex that functions in quaternary protein folding.

## Results

### The RPAP3 C-ter domain co-evolved with PIH-domain proteins

Although extensive structural studies have been performed with R2TP proteins^2^, the structure and function of RPAP3-Cter is unknown. This domain is absent in the *S. cerevisiae* homolog of RPAP3, and we thus performed an evolutionary analysis (Fig. 1c). Besides RPAP3, the human genome encodes two proteins harboring a RPAP3-Cter domain: SPAG1, which contains three TPR domains, and CCDC103, which has a N-terminal dynein attachment domain. Proteins with both an RPAP3-Cter and a TPR domain occur in all eukaryotic lineages (Fig. 1c; blue dots in the RPAP3_C column). A single such protein was likely present at the root of Eukaryotes, and a duplication event having occurred between Amoebozoa and Opisthokonta (Fungi and Metazoa) generated RPAP3 and SPAG1. The human genome encodes four proteins with PIH domains: PIH1D1-3 and DNAAF2 (also known as Kintoun). DNAAF2 associates with DYX1C1^29^, a CS- and TPR- containing protein that we included in our analysis. Most eukaryotic lineages encode three PIH-proteins and one DYXC1 ortholog. These proteins are thus of ancient origin, as previously noted^30^. Interestingly, the duplication of the RPAP3 ancestor is mimicked by a similar contemporary duplication of the ancestor of PIH1D1, which generated PIH1D1 and PIH1D2. Note that neither RUVBL1/2 nor other HSP90 co-chaperone were duplicated (Fig. 1c). Altogether, this analysis indicates that the ancestral form of RPAP3 had both TPR and RPAP3-Cter domains, suggesting an important function for this domain. This protein further co-evolved with the ancestor of PIH1D1, pointing to a link with R2TP.

### The NMR structure of RPAP3-Cter reveals a helical 3D fold

To get more information on RPAP3-Cter, we performed structural studies. We expressed fragments 535–665 and 547–665 of human RPAP3. Only the largest domain, i.e., RPAP3_535-665_, was soluble after expression and purification from *E. coli* (Supplementary Fig. 1A, B). Using NMR, the intensity and distribution of peaks in the ^1^H-^15^N HSQC spectrum revealed a properly folded domain (Supplementary Fig. 1C). We obtained a well-resolved set of 20 water-refined structures (Table 1), revealing that this domain is composed of 8 α-helices that pack together to form a globular object (Fig. 1d–f). Interestingly, the residues 541–548 form a loop that protects several hydrophobic amino-acids from solvent, including Leu542, Pro543, Ile545, and Pro546 (Supplementary Fig. 1D). This loop contributes to the solubility of RPAP3-Cter since its truncation in RPAP3_547-665_ leads to protein aggregation. We submitted our structure to the DALI server to search for potential structural homologs. No strong candidate could be identified using a top DALI Z-score below 8, with a mean RMSD and identity percentage of 3.6 Å and 9.7%, respectively. We concluded that RPAP3-Cter adopts a 3D fold, linked to an uncharacterized biological function.

**Table 1:** NMR and refinement statistics for top-20 RPAP3_535-665_ structures

	RPAP3_535-665_
**NMR distance and dihedral constraints**	
Distance constraints	
Total NOE	3707
Intra-residue	855
Inter-residue	2852
Sequential (|*i* – *j*| = 1)	849
Medium-range (|*i* – *j*| ≤ 4)	1033
Long-range (|*i* – *j*| ≥ 5)	970
Total dihedral angle restraints	230
ϕ	110
ψ	120
**Structure statistics after AMBER refinement**	
Violations	
Distance constraints (Å)	0.10 ± 0.03
Dihedral angle constraints (°)	6.70 ± 1.94
Max. dihedral angle violation (°)	9.99
Max. distance constraint violation (Å)	0.14
Violation occurrences	
Distances constraints ( > 0.2 Å)	0
Dihedral angle constraints ( > 5°)	1.30 ± 1.26
R.m.s. deviations from idealized geometry	
Bond lengths (Å)	0.013 ± 0.00
Bond angles (°)	1.85 ± 0.05
R.m.s. deviation to best structure^a^ (Å)	
All heavy atoms	1.33 ± 0.29
All backbone atoms	0.98 ± 0.38
Heavy atoms in secondary structures	0.94 ± 0.07
Backbone atoms in secondary structures	0.37 ± 0.05

^a^Pairwise r.m.s. deviation was calculated among 20 refined structures

### RPAP3-Cter associates with RUVBL1/2 and some R2TP clients

**Fig. 2:**
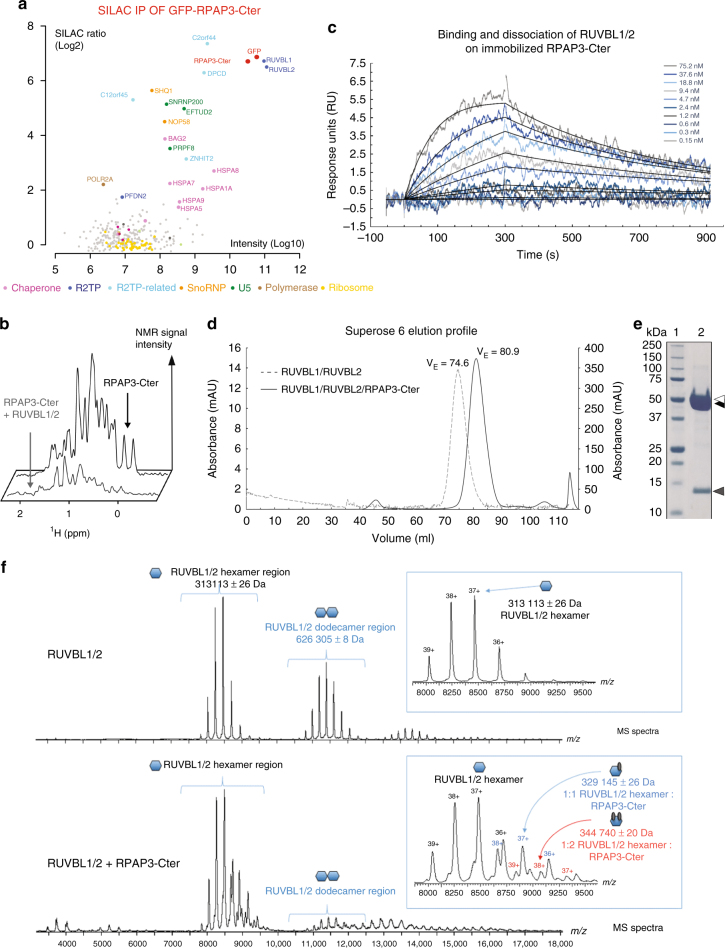
RPAP3-Cter interacts with RUVBL1/2 hexamers. **a** SILAC proteomic analysis of RPAP3-Cter. The graph depicts the proteins identified in anti-GFP immuno-precipitates of HeLa cells expressing GFP-RPAP3-Cter. Each dot is a protein and the color code is indicated below the graph. *X*-axis: protein abundance (Log10 of signal intensity); y-axis: enrichment over a control IP (Log2 of SILAC ratio). **b** NMR interaction analysis of RPAP3-Cter with recombinant RUVBL1/2 complex. The graph depicts 1D NMR METHYL-SOFAST-HMQC spectra in the methyl region of ^13^C-labeled RPAP3-Cter alone (top lane) or mixed with recombinant RUVBL1/2 complex (bottom lane). Intensity of the NMR signal (arbitrary units, *Y*-axis) is plotted against the ^1^H chemical shift (in ppm, *X*-axis). **c** SPR binding assays of RPAP3-Cter with RUVBL1/2. The graph depicts the response upon injecting the RUVBL1/2 complex (t = 0 s), or upon washing (t = 300 s), on immobilized RPAP3-Cter. *X*-axis: time (s); *Y*-axis: response (arbitrary units). These data have been obtained with the same batch of RUVBL1/2 complex as in the control experiment (Fig. S2E, F). **d** Chromatographic analysis of the RUVBL complexes. The graph depicts the chromatograms of purified RUVBL1–RUVBL2 (dashed gray, left Y axis) or RUVBL1-RUVBL2-RPAP3-Cter (black line, right Y axis), on a Superose 6 16/70 XK. X-axis: elution volume; Y-axis: absorbance.  **e** Electrophoresis of the purified RUVBL1–RUVBL2–RPAP3–Cter complex. The gel shows the peak fraction of the complex eluted from the column (black line in d), with a purity estimated to ~95 %. Lane 1: Precision Plus Protein Unstained Standards (Biorad); Lane 2: denatured RUVBL1–RUVBL2–RPAP3–Cter complex. Black and white arrows: RUVBL1 and RUVBL2 (52 and 53 kDa, respectively); gray arrow: RPAP3-Cter (15 kDa). **f** Native mass spectrometry analysis of recombinant RUVBL complexes. The upper mass spectrum presents the purified RUVBL1/2 complex. The bottom mass spectrum presents the same complex after addition of RPAP3-Cter. Y-axis: signal intensity; X-axis: m/z. Insets: zoom over the 8000–9000 m/z region. Schematics depict the complex observed. Blue: RUVBL proteins; red: RPAP3-Cter

To get insights into the function of RPAP3-Cter, we characterized its partners by performing a proteomic analysis in human cells. We fused this domain to GFP and stably expressed it in HeLa cells using site-specific Flp-In integration. Following differential labeling of GFP-RPAP3-Cter and control cells with isotopic amino-acids (SILAC), whole cell extracts were immuno-precipitated (IP) with anti-GFP antibodies and pellets were subjected to quantitative mass-spectrometry analysis (Fig. 2a). About 20 proteins associated with RPAP3-Cter with high abundance (intensity) and specificity (SILAC ratio). The top hits were RUVBL1/2, while the others belonged to several classes: (i) known R2TP clients (SHQ1 and NOP58 for snoRNPs, PRPF8, and other U5 proteins for snRNAs, POLR2A for RNA polymerase II); (ii) known R2TP cofactors (ZNHIT2) and/or RUVBL1/2 partners (C12ORF45, C2ORF44, DPCD)^6,17,21^; (iii) PAQosome subunit (PFDN2)^2^; and (iv) chaperones (HSP70 isoforms and its regulator BAG2). Overall, these data suggest that RPAP3-Cter may play an important role in interacting with RUVBL1/2 and some R2TP clients.

### RPAP3-Cter directly binds RUVBL1/2

We then performed pairwise yeast two-hybrid assays (Y2H) between RPAP3-Cter and its putative partners (Supplementary Fig. 2A). We detected a positive signal with RUVBL2, but not with the other tested proteins. To determine whether this interaction is direct, we used recombinant proteins for in vitro binding assays. RUVBL1/2 were co-expressed in *E. coli* and they purified predominantly as a dodecamer (see below). We then assessed the binding to RPAP3-Cter using NMR spectroscopy. We took advantage of the ^13^C-labeling to monitor the effect of RUVBL1/2 on the methyl groups of RPAP3-Cter. Indeed, methyl groups are able to provide strong signals even at low protein concentrations and/or basic pHs^31^. The NMR signal of ^13^C-labeled RPAP3-Cter decreased dramatically in presence of RUVBL1/2 hetero-multimers (Fig. 2b), while only minor effects were seen with RUVBL1 or RUVBL2 alone (Supplementary Fig. 2B). Next, we used SPR and observed robust interactions between RPAP3-Cter and RUVBL1/2 heteromultimers (Fig. 2c), with a K_D_ of 4.2 nM in a 1:1 interaction model calculation (Table 2). These data demonstrated that RPAP3-Cter binds heteromeric forms of RUVBL1/2.

**Table 2:** RUVBL1/2:RPAP3–Cter affinity and kinetic interaction parameters determined by SPR

His_RUVBL1_Flag_RUVBL2 interaction with immobilized RPAP3-Cter			
K_D_ (M)	k_d_ (s^−1^)	k_a_ (M^−1^ s^−1^)	*n* ^a^
4.18×10 ^−9^ ± 1.96×10 ^−9^	1.12×10 ^−3^ ± 2.58×10 ^−4^	2.97×10 ^5^ ± 6.35×10 ^4^	3

^a^*n* stands for triplicate

RUVBL1/2 cycle between hetero-hexamers and hetero-dodecamers^20^, and we thus addressed the stoichiometry of the interaction. First, we co-expressed RPAP3-Cter with RUVBL1/2 in *E. coli* and analyzed the complex by gel-filtration (Fig. 2d, e). The eluting volumes of RUVBL1/2 alone and RUVBL1/2:RPAP3-Cter corresponded to molecular masses of dodecameric and hexameric forms of RUVBL1/2, respectively. Next, we performed mass spectrometry analysis under non-denaturing conditions (native MS), of either RUVBL1/2 alone, or RUVBL1/2 co-purified with recombinant RPAP3-Cter (Fig. 2f). In the free state, RUVBL1/2 formed hetero-hexameric and hetero-dodecameric complexes, but hexameric forms became largely dominant upon RPAP3-Cter binding. Note also that one hexamer can bind multiple RPAP3-Cter domains. These data indicate that RPAP3-Cter binds predominantly RUVBL1/2 hetero-hexamers.

Next, we analyzed the binding of RPAP3-Cter to RUVBL mutants altered in their ATPase cycle. For this, we turned to LUMIER interaction experiments. In this quantitative co-IP assay, the prey and bait proteins are respectively fused to Renilla luciferase (RL) and FLAG-tagged Firefly luciferase (3xFLAG-FFL). Following transient expression in human HEK293 cells, the bait is IP’ed with anti-FLAG antibodies, or without antibodies as control. The RL and FFL luciferase activities are then measured in the input and pellet, and the co-IP efficiency is expressed as the percent of prey co-immuno-precipitated, relative to that of the bait, providing a direct measurement of binding efficiencies (Supplementary Fig. 2D). In these experiments, we used canonical mutations of AAA + ATPases^32^, expected to prevent nucleotide binding (K to M mutant in the Walker A domain), nucleotide hydrolysis (E to Q in the Walker B-domain), or coupling between adjacent monomers (R to E in the Arg finger). The effect of several of these mutations was previously validated using the *Chaetomium thermophilum* orthologs of RUVBL1 and RUVBL2^20^. Interestingly, while the K-M and R-E mutations had little effects on the association of either RUVBL1 or RUVBL2 with RPAP3-Cter, the E-Q mutations diminished binding (Supplementary Fig. 2D). Taken together, these data indicated that RPAP3-Cter made a direct, high-affinity interaction with hexameric RUVBL1/2. This interaction did not require nucleotide and was weakened in mutants deficient in ATP hydrolysis.

### RPAP3-Cter binds R2TP clients through RUVBL1/2

**Fig. 3:**
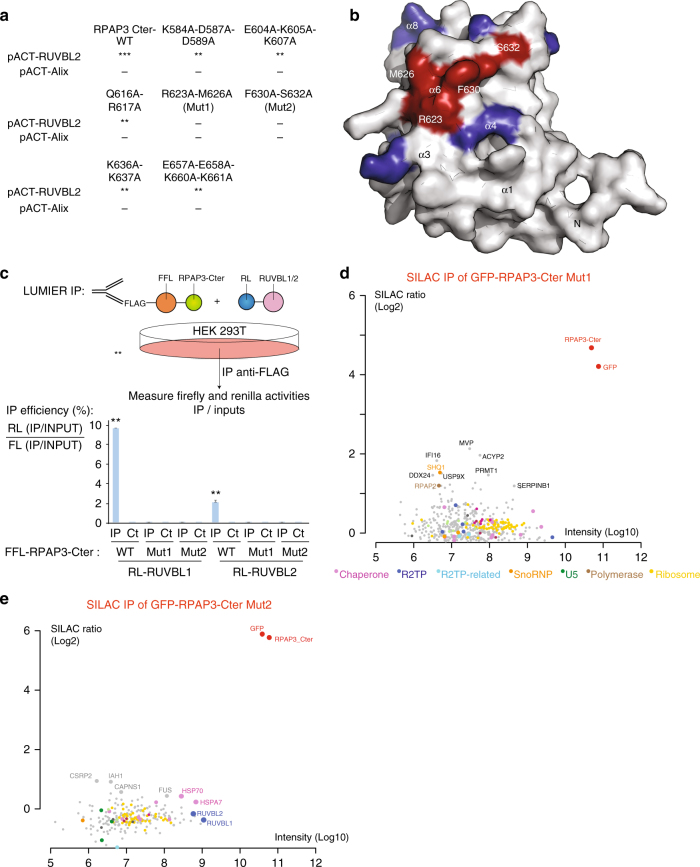
The RPAP3 C-terminal domain interacts with R2TP clients via RUVBL1/2 multimers. **a** Yeast two-hybrid analysis of interactions between RUVBL2 and RPAP3-Cter mutants. Alix is a negative control. ***: strong interaction; **: medium; *: weak; −: no interaction. **b** Molecular surface representation of RPAP3-Cter structure by specifying the location of the mutants that lost interaction with RUVBL1/2. **c** LUMIER assay showing the in vivo interaction between RPAP3-Cter and RUVBL1/2 mutant proteins. Top panel: schematic representation of the assay. Bottom panel: graph plotting the IP efficiency of the indicated proteins. The values are the IP efficiencies of the co-precipitation of the RL fusion proteins (IP/Input), normalized by the IP/Input values obtained with the anti-FLAG IP of the 3xFLAG-FFL fusion protein. Error bars: standard deviation. Stars: values significantly greater than six-times the mean value obtained in the control IPs without anti-FLAG antibody (Ct). **p-value < 0.001 (Z-test). **d,e** SILAC proteomic analysis of the partners of RPAP3-Cter-Mut1 and RPAP3-Cter-Mut2, respectively. Legend as in Fig. 2a

Our proteomic analysis of RPAP3-Cter showed that it binds not only RUVBL1/2, but also a range of R2TP clients. To test whether these latter interactions depend on RUVBL1/2, we generated a series of RPAP3-Cter mutants. Structure-guided analysis selected solvent-exposed residues potentially involved in RUVBL1/2 interaction (Supplementary Fig. 3A, B). Alanine mutants were screened by Y2H assays, and two of them indeed lost interaction with RUVBL2 (Fig. 3a): R623A-M626A (Mut1) and F630A-S632A (Mut2). Both mutants localized similar to wild-type RPAP3-Cter (Supplementary Fig. 3C), suggesting no major alteration. The mutated residues are conserved across evolution (Supplementary Fig. 3D). They were located next to each other in the 3D structure of RPAP3-Cter (Fig. 3b), suggesting that they may constitute a conserved binding site for RUVBL1/2. LUMIER interaction assays confirmed the Y2H data, as both RPAP3-Cter Mut1 and Mut2 lost interaction with RUVBL1 and RUVBL2 (Fig. 3c). Next, we performed SILAC quantitative proteomic analyses of the RPAP3-Cter mutants. Remarkably, both mutants lost all of the RPAP3-Cter partners identified previously (Fig. 3d, e), with the exception of SHQ1 that remained weakly bound to RPAP3-Cter Mut1. Taken together, these data suggest that RUVBL1/2 are required for the association of RPAP3-Cter with its other partners.

### Identification of RPAP3-like/PIH-like complexes

**Fig. 4:**
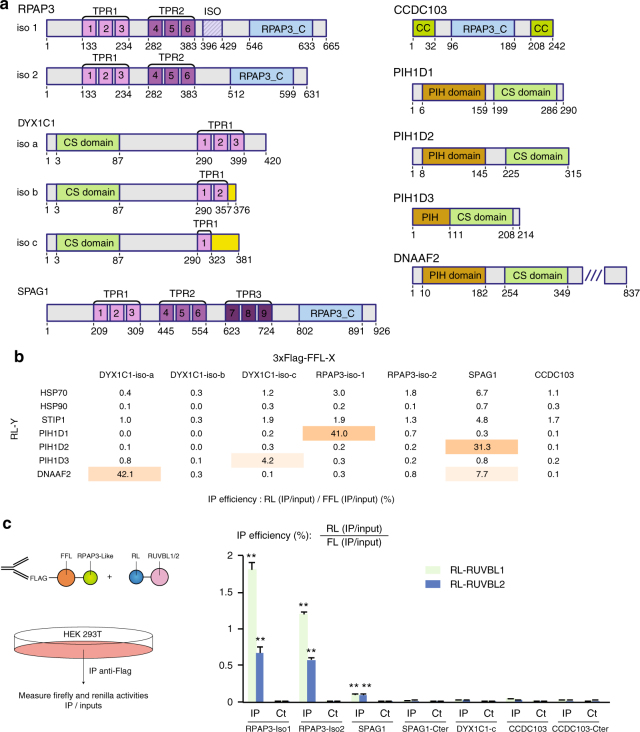
RPAP3-like and PIH1-like proteins interact with each other. **a** Architecture of the human proteins containing a RPAP3-Cterminal domain (RPAP3-C), or a PIH domain (PIH). Coiled-coil (CC), CHORD-containing proteins and SGT1 domain (CS) and TPR domains (TPR) are also indicated. Different splicing isoforms of RPAP3 and DYX1C1 are shown, with their variable domains in hatched violet (RPAP3), or yellow (DYX1C1). **b** Summary of pairwise LUMIER interaction assays between the indicated proteins. The values are the efficiencies of the co-precipitation of the RL fusion proteins (IP/Input), expressed in percent of the efficiencies obtained with the 3xFLAG-FFL fusions. *p*-values are shown in Supplementary Fig. 4C. **c** LUMIER interaction assays between RL-RUVBL1/2 and RPAP3-like proteins tagged with 3xFLAG-FL. Legend as in Fig. 3c. Stars: values significantly greater than six-times the mean value obtained in the control IP (Ct). **p-value < 0.001 (*Z*-test)

As described above, the human genome encodes two other proteins bearing RPAP3-Cter-like domains, SPAG1 and CCDC103, and three others containing PIH domains, PIH1D2, PIH1D3 and DNAAF2 (Fig. 1c, Fig. 4a). This suggested that these proteins could form R2TP-like complexes, by associating to each other and with RUVBL1/2 through their RPAP3-Cter-like domains. To test this possibility, we first performed systematic pairwise LUMIER co-IP assays between RPAP3-like and PIH-like proteins (Fig. 4b). In these tests, we also added DYX1C1 because this protein associates with DNAAF2^29^ and bears some structural features of RPAP3 and PIH1D1 (e.g., TPR and CS domains), although it lacks PIH and RPAP3-Cter domains. We also included the known splicing isoforms of DYX1C1 and RPAP3, including the RPAP3 isoform 2 that does not interact with PIH1D1^33^. Finally, we added HSP70, HSP90, and STIP1, a factor promoting the transfer of clients from HSP70 to HSP90^34^. These proteins were fused at their N-termini to Renilla luciferase or to 3xFLAG-Firefly luciferase. The plasmids were transiently transfected in HEK293 cells, and LUMIER IPs were performed (Fig. 4b). As above, the interaction strength was expressed as the fraction of co-IP’ed prey protein normalized to that of the bait. Negative controls included four unrelated proteins, which were tested against all the RPAP3-like and PIH-like proteins (Supplementary Fig. 4A). This allowed us to rigorously identify specific interactions (see Methods). These experiments revealed that all the TPR-containing proteins interacted with HSP70, HSP90 and STIP1, although the co-IP efficiency was low (Fig. 4b and S4B). This could be due to a low affinity or to the fact that HSPs have many partners that potentially compete with baits in extracts. Most interestingly, we uncovered three strong associations between RPAP3-like and PIH-like proteins (30–40% of co-IP efficiencies): RPAP3-iso-1 with PIH1D1, DYX1C1-iso-a with DNAAF2, and SPAG1 with PIH1D2. Two other strong interactions were found, between DYX1C1-iso-c and PIH1D3, and between DNAAF2 and SPAG1, although the IP efficiencies were 5–10 times less than for the previous couples (4–8% of co-IP efficiencies). Finally, a number of weak but significant interactions were also found (Fig. 4b and S4C), suggesting that additional PIH1D1-like/RPAP3-like pairs may also form, albeit at low efficiencies.

### PIH1D2 facilitates the binding of SPAG1 to RUVBL1/2

**Fig. 5:**
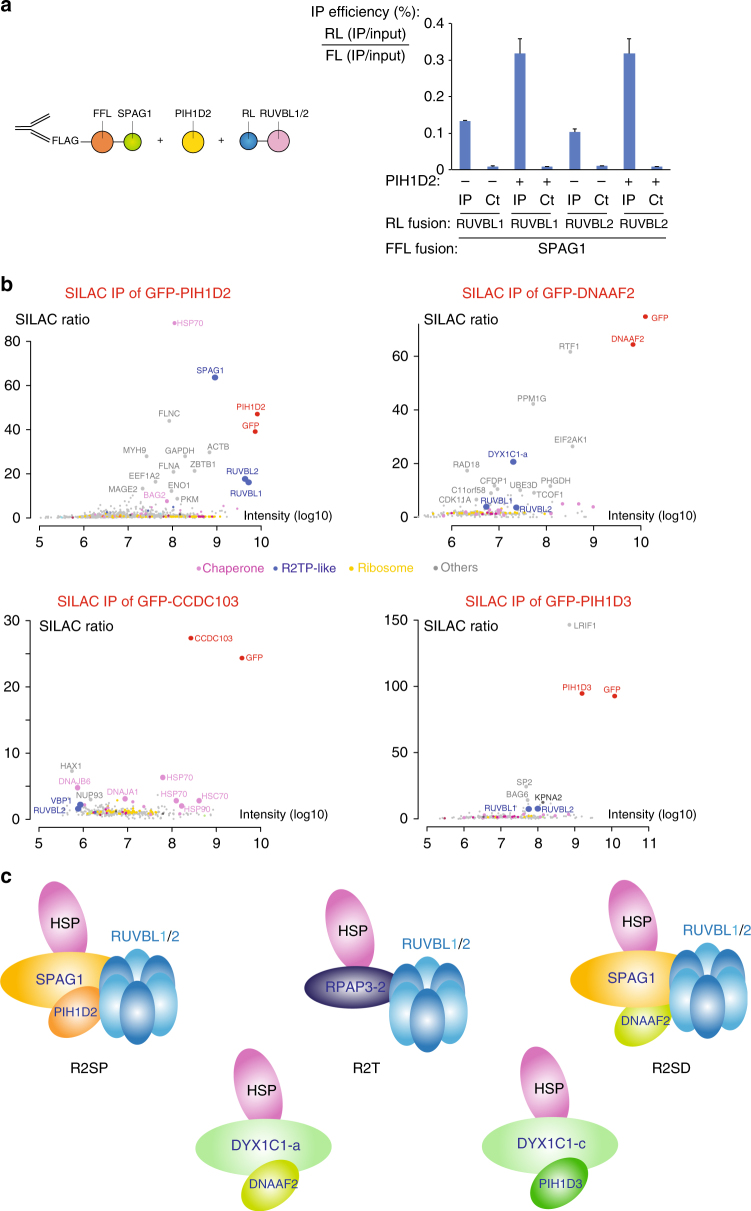
Identification of R2TP-like complexes. **a** LUMIER interaction assays between SPAG1 and RUVBL1/2. Legend as in Fig. 3c excepted that an untagged PIH1D2 was co-expressed (lanes+), or SMD1 as control (lanes−). **b** SILAC proteomic analysis of the partners of GFP-PIH1D2, GFP-DNAAF2, GFP-CCDC103, and GFP-PIH1D3, performed in HeLa cells. Legend as in Fig. 2a. The color code is indicated between the graphs. **c** Models of possible R2TP-related complexes

We then tested whether SPAG1 and CCDC103 would bind RUVBL1/2, also using LUMIER assays (Fig. 4c). SPAG1, RPAP3-iso-1 and iso-2 interacted with both RUVBL proteins, while CCDC103 did not. Surprisingly however, the isolated C-terminal domain of SPAG1 failed to interact with RUVBL1 or RUVBL2. The C-terminal domains of RPAP3, SPAG1, and CCDC103 have an overall identity of only 25%, and the sequence differences may modulate their affinity for RUVBL1/2, from a high affinity binding for RPAP3-Cter, to a low or no binding for SPAG1-Cter and CCDC103-Cter. Indeed, the amino-acids required for the binding of RPAP3-Cter to RUVBL1/2 are not strictly conserved in SPAG1 and CCDC103 (Supplementary Fig. 3B). Since the full-length SPAG1 protein bound better to RUVBL1/2 than the isolated SPAG1-Cter domain, we further tested whether its PIH1D2 partner would enhance binding. We repeated the LUMIER assay in presence of co-expressed, untagged PIH1D2 (Fig. 5a). Indeed, PIH1D2 increased the binding of SPAG1 to RUVBL1 and RUVBL2 by nearly 3-fold, achieving a binding efficiency similar to that of full-length RPAP3. Taken together, these data indicate that SPAG1 binds RUVBL1/2 and that the binding determinants likely include several regions within SPAG1 and PIH1D2.

This result prompted us to test whether the PIH-like proteins would interact with RUVBL1/2. LUMIER assays indicated that significant interactions could be detected (Supplementary Fig. 4D), but at a low level (5–20 fold weaker than with RPAP3).

### Human cells contain several complexes related to R2TP

The LUMIER assays indicate that SPAG1 can interact with PIH1D2 and RUVBL1/2, possibly forming a complex similar to R2TP. To obtain direct evidence that this complex exists, and to determine whether additional R2TP-like complexes may form, we turned to quantitative SILAC proteomics. We fused GFP to PIH1D2, PIH1D3, DNAAF2, and CCDC103, stably expressed the fusions in HeLa cells and used them as baits in proteomic experiments (Fig. 5b and Supplementary Data 1). We did not find any partner for GFP-CCDC103. For GFP-PIH1D3, we found a strong association with LRIF1, a nuclear factor enriched in testis. Only minute amounts of RUVBL1/2 were detected, in agreement with the weak interaction observed in the LUMIER assays. DYX1C1-iso-c was also not detected, possibly because of its low abundance in HeLa cells (Gtex dataset^35^). For GFP-DNAAF2, we found a strong association with DYX1C1-iso-a, and again a weak binding to RUVBL1 and RUVBL2 that agreed with the LUMIER assays (Fig. 4b and S4C). In the GFP-PIH1D2 IP, the three most prominent proteins were SPAG1, RUVBL1 and RUVBL2, all found with both a high specificity and abundance (Fig. 5b). HSP70 and its regulator BAG2 were also significantly enriched. This indicated the existence of an R2TP-like complex containing PIH1D2, SPAG1, RUVBL1/2, which associated with HSP70 chaperones. The other GFP-PIH1D2 partners found in this experiment were involved in a variety of processes ranging from metabolism to RNA processing, and may represent clients of this chaperone system. No prefoldin nor prefoldin-like proteins were detected, but we noted that an interaction of SPAG1 with WDR92 has been previously described^21^.

Collectively, the LUMIER and proteomic data thus defines three types of complexes related to R2TP (Fig. 5c). A first complex is composed of RPAP3-iso-2 in association with RUVBL1/2. This complex is identical to the canonical R2TP except that it lacks PIH1D1, and was thus named R2T. A second complex comprises SPAG1, PIH1D2 and RUVBL1/2. This complex shares an organization similar to R2TP and was named R2SP. Another related complex could form with SPAG1, DNAAF2 and RUVBL1/2 (i.e., an R2SD complex). However, this complex remains hypothetical since it was detected in LUMIER but not in proteomic experiments. Finally, we observed two heterodimers containing a PIH-like protein associated with a DYX1C1 isoform: DNAAF2/DYX1C1-iso-a and PIH1D3/DYX1C1-iso-c. These interactions were previously reported^29,36^, but the isoform specificity of DYX1C1 was not known.

The occurrence and composition of these complexes is corroborated by our evolutionary analyses (Fig. 1c). The parallel duplication leading to RPAP3 and SPAG1 on one side, and PIH1D1 and PIH1D2 on the other, is consistent with their respective incorporation into R2TP and R2SP. Similarly, some species contain an RPAP3 ortholog but lack a PIH1D1/PIH1D2 gene, thus mirroring the existence of R2T in human cells. The frequent co-occurrence of DYX1C1 with either DNAAF2 or PIH1D3 is also consistent with the association observed for the human proteins.

### R2TP-like components are enriched in testis

To gain insights into the function of these R2TP-related complexes, fluorescent microscopy was performed with the stable cells expressing the GFP-tagged proteins. These proteins localized to different cellular areas, suggesting specialized functions (Supplementary Fig. 5). Interestingly, DYX1C1-iso-c was nuclear and concentrated in punctate structures, as did its partner PIH1D3. It is also worth noting that RPAP3-iso-2 was mainly nuclear while RPAP3-iso-1 was mainly cytoplasmic. Thus, alternative splicing determines not only the partners but also the localization of these proteins.

Next, we examined existing data to determine in which tissues these factors are expressed (Gtex dataset^35^; Supplementary Fig. 6A). The expression patterns of RUVBL1, RPAP3, and PIH1D1 looked similar to each other, with a rather ubiquitous expression. DNAAF2 and SPAG1 were also broadly expressed, but with a moderate enrichment in testis. Interestingly, PIH1D2, PIH1D3, and RUVBL2 were highly expressed in testis, suggesting an important role in this organ.

### PIH1D2 has both ubiquitous and testis-enriched partners

**Fig. 6:**
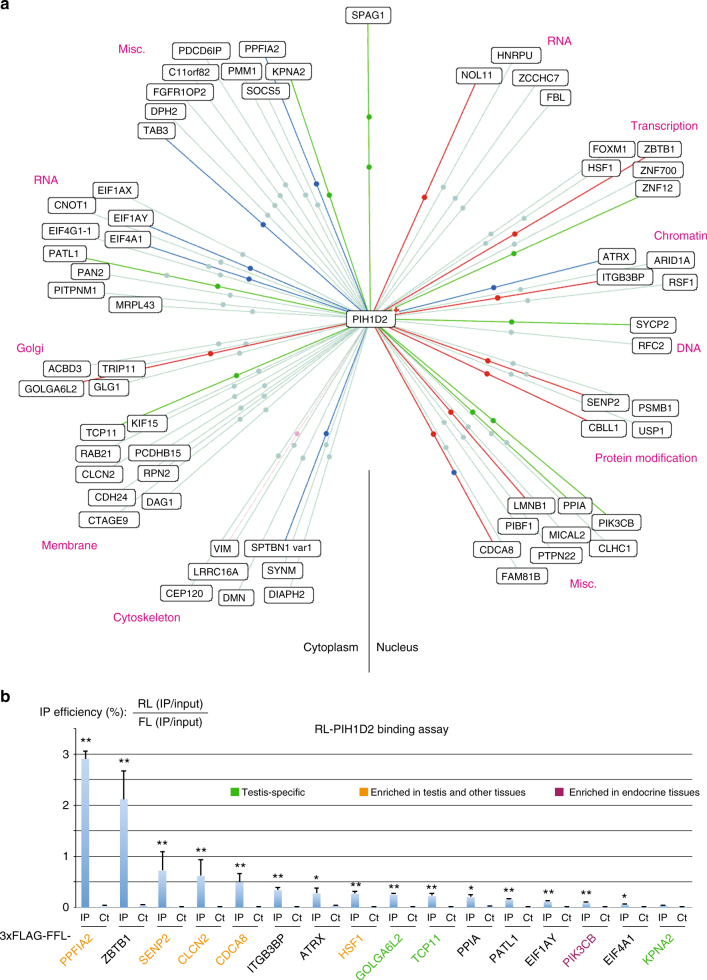
Identification of PIH1D2 partners. **a** Results of yeast two-hybrid screens using human PIH1D2 as bait and performed with human libraries from lung carcinoma cell lines and testis. The color of the lines indicate the strength of the Y2H interaction (PBS score). a red; b dark blue; c green; d light blue. Lines with two dots indicate that the prey was found in the two libraries. **b** Validation of the hits found in the yeast two-hybrid screens by LUMIER co-IP assays. The graph depicts the results of LUMIER co-IP assays performed with the indicated proteins. Error bars: standard deviation. Stars: values significantly greater than six-times the mean value obtained in the control IPs without anti-FLAG antibody (Ct). *p-value < 0.05; **p-value < 0.001 (Z-test)

To characterize the function of R2SP, we first searched for partners by performing yeast two-hybrid screens using PIH1D2 as bait. Given its expression pattern, we screened two human libraries, from lung cancer cell lines and from testis (Fig. 6a). The screens revealed a total of >60 potential partners (46 from the lung and 32 from testis; Fig. 6a and Supplementary Data 2). Nine proteins were found in both libraries, including SPAG1, indicating the high quality of the screens. These PIH1D2 partners are involved in a variety of functions, ranging from DNA metabolism, transcription and RNA processing, and up to cytoskeletal organization, membrane-related processes and trafficking.

Next, we selected 16 proteins to test LUMIER assays and could validate most of them (Fig. 6b). Taken together, these data demonstrated that PIH1D2 has a range of partners involved in a variety of processes. Some partners were enriched in testis and others were ubiquitous (Gtex dataset^35^, Fig. 6b).

### R2SP facilitates quaternary protein folding

**Fig. 7:**
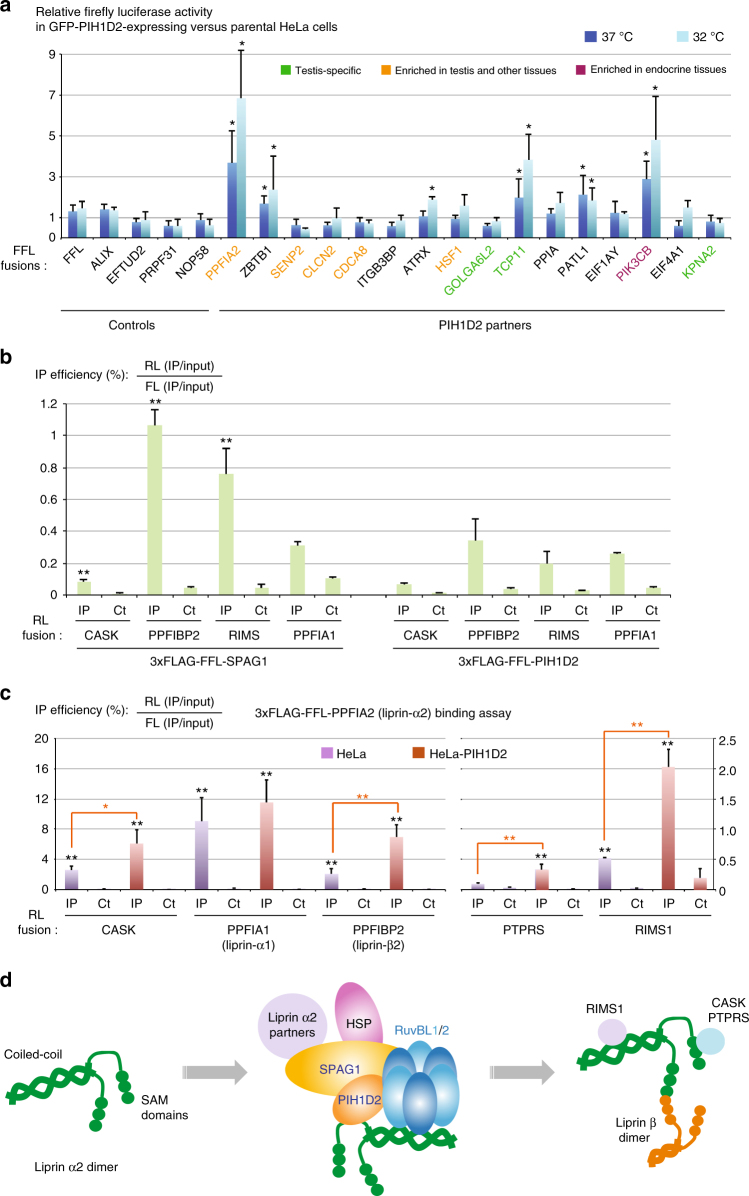
The R2SP complex promotes the stabilization of its clients and the assembly of liprin-α2 complexes. **a** R2SP enhances expression of some of its clients. The graph depicts the relative expression levels of the indicated FFL-fusion proteins in HeLa cells expressing PIH1D2, vs. parental HeLa cells not expressing it. Dark blue: experiment performed at 37 °C; light blue: experiment performed at 32 °C. Values are normalized by the mean of controls (left); error bars: standard deviation. **p-value < 0.02 with a *t*-test involving all the control samples (*n* > = 3). **b** Binding of PPFIA2-related proteins to SPAG1 and PIH1D2. The graph depicts LUMIER interaction assays between the indicated proteins. Error bars: standard deviation. Stars: values significantly greater than six-times the mean value obtained in the control IPs without anti-FLAG antibody (Ct). *p-value < 0.05; **p-value < 0.001 (Z-test). **c** R2SP promotes association of liprin-α2 (PPFIA2) with its partners. The graph depicts LUMIER interaction assays between PPFIA2 and its partners, in HeLa cells expressing or not PIH1D2. Legend as in Fig. 3c, with single black stars indicating a *p*-value < 0.05, and double black stars a *p*-value < 0.001 (Z-test comparing values of the FLAG IPs with six-times the mean value obtained in the control IP). Orange stars: comparison of HeLa and HeLa-PIH1D2 cells. Error bars: standard deviation. *p-values < 0.05; **p-values < 0.005 (T-test). **d** Assembly of liprin-α2 complexes by R2SP

Next, we tested whether R2SP has a chaperone activity toward its partners. Since PIH1D2 is poorly expressed in HeLa cells, in contrast to the other components of R2SP (Supplementary Fig. 6B), we generated HeLa cells stably expressing GFP-PIH1D2, and thus having a fully assembled R2SP complex (see Fig. 5b). To measure its chaperone activity, we fused to Firefly luciferase the PIH1D2 partners previously identified, and we transiently transfected them in HeLa-PIH1D2 and parental HeLa cells. Remarkably, five fusions were significantly more expressed in presence of GFP-PIH1D2, reaching a threefold increase in one case (Fig. 7a). These were PPFIA2 (liprin-α2), ZBTB1, TCP11, PATL1 and PIK3CB. In contrast, the expression of a broad series of control proteins, including unrelated factors (Alix, FFL) or known R2TP-substrates (EFTUD2, PRPF31, NOP58), was identical in both cell lines (Fig. 7a). Next, we repeated the experiments at 32 °C, the optimal temperature of testis where PIH1D2 is highly expressed. The effects of R2SP on proteins levels were generally more important at this temperature. Altogether, this suggested that R2SP enhanced expression of some of its partners and had a stronger effect at the testis temperature.

Since R2TP is involved in the assembly of its target complexes^2^, we hypothesized that R2SP could do the same. To test this, we focused on liprin-α2 (PPFIA2). Indeed, this is the strongest binder of PIH1D2 and it is also the most sensitive to the presence of PIH1D2. In addition, liprins are important scaffolding molecules that bring together a diverse set of factors in order to control cell adhesion, cell migration, and organization of the active synaptic zone^37^. Liprins possess a long coiled-coil domain at their N-terminus, followed by a linker and three Sterile Alpha Motif domains (SAM). The coiled-coil domain of liprin-α2 can dimerize or heteromerize with liprin-α1 and α3^38^. This domain also binds several proteins, including RIMS1, a protein involved in the docking of exocytic vesicles^37,39^. The SAM domain of liprin-α2 interacts with the kinase CASK, as well as with the tyrosine phosphatases LAR (PTPRF), PTPRD and PTPRS^38^. In addition, it can simultaneously interact with liprin-β to organize higher order molecular assemblies^39^.

We first determined whether R2SP could interact with the partners of liprin-α2. We fused liprin-α1, liprin-β2 (PPFIBP2), CASK and RIMS1 to Renilla Luciferase and to flagged Firefly Luciferase, and performed LUMIER assays with R2SP subunits (Fig. 7b). Indeed, CASK, liprin-β2 and RIMS1 interacted with SPAG1. Next, we tested whether R2SP would facilitate the association of liprin-α2 with its partners. FLAG-FFL-liprin-α2 was transfected in HeLa and HeLa-PIH1D2 cells, and LUMIER IPs were performed with RL-tagged liprin-α2 partners. Of note, a larger amount of the FLAG-tagged liprin-α2 plasmid was transfected in HeLa cells to compensate for its lower level of expression in this cell line, such that a similar ratio of bait over preys was obtained in HeLa and HeLa-PIH1D2 cells. The efficiency of co-precipitation of liprin-α1 by liprin-α2 was similar in HeLa and HeLa-PIH1D2 cells. In contrast, liprin-β2, CASK, RIMS1 and PTPRS were all co-precipitated more efficiently by liprin-α2 in HeLa-PIH1D2 cells (2.3–3.9 fold; Fig. 7c). These data demonstrate that the presence of PIH1D2, and thus of a full R2SP complex, promotes the association of liprin-α2 with several of its targets. This indicates that R2SP is involved in quaternary protein folding.

## Discussion

R2TP is a conserved HSP90 co-chaperone that is involved in the assembly of key cellular complexes^2^. *S. cerevisiae* and human R2TP share a similar organization but striking differences distinguish their RPAP3/Tah1p subunit. Human RPAP3 contains two central TPR domains that bind HSP70 and HSP90, and we show here that its C-terminal domain adopts a helix-bundle fold and bind RUVBL1/2 hexamers. In contrast, *S. cerevisae* Tah1p is six times smaller than human RPAP3 and consists of a single short TPR domain that functions as an adapter between Hsp90 and Pih1p^15,25^. In particular, Tah1p lacks the RPAP3-Cter homology domain and consequently, the Rvb ATPases are mainly recruited by Pih1p in yeast^26,28^. This structural difference likely translates into different functions. In *S. cerevisiae*, *TAH1* knock-out displays much milder phenotypes than *PIH1*^40^, while Drosophila and mouse *RPAP3* are essential genes (^41^ and unpublished observations).

The PIH domain of PIH1D1 recruits some client proteins via a phosphopeptide-binding pocket that binds DSpDD/E motifs^14,15^. We show here that RPAP3-Cter binds a large number of R2TP clients and is thus also involved in client recruitment. Interestingly, binding of these clients is lost in RPAP3 mutants that no longer bind RUVBL1/2. RPAP3-Cter could make cooperative interactions with RUVBL1/2 to bind the clients, or it may only bind the ATPases, which would in turn recruit the clients. An interesting possibility would be that RPAP3-Cter maintains the ATPases in a conformation suitable for client binding, in a manner analogous to CDC37 for HSP90^42^. In agreement with this idea, RPAP3-Cter has different affinities for RUVBL mutants locked at different stages of their ATPase cycle.

It was recently proposed that the RUVBLs cycle between single and double ring structures and that this may give them chaperone activity ^16^. Interestingly, dimerization of the hexameric rings involves their DII domains, and a recent cryo-EM structure of the *S. cerevisiae* R2TP complex revealed that the Tah1p:Pih1p heterodimer also associates with this domain. The position of Tah1p:Pih1p thus appears ideal to regulate the formation of double ring structures. Given the very different interaction of human RPAP3-PIH1D1 with RUVBL1/2, it will be interesting to determine whether a similar structural arrangement is conserved in human R2TP.

SPAG1 has an organization similar to RPAP3, with three TPR domains preceding the RPAP3-Cter-like domain. We show here that SPAG1 forms an R2TP-like complex with RUVBL1/2 and PIH1D2, which we termed R2SP. Our two-hybrid screen indicates that a short region downstream the third TPR of SPAG1 is involved in PIH1D2 binding (Supplementary Data 2). Interestingly, the difference between the two isoforms of RPAP3 occurs at a similar location, and this region also determines binding to PIH1D1 (ref. ^33^ and Fig. 4b). This is reminiscent of the binding of Tah1p to Pih1p, where a short C-terminal region of Tah1p, located immediately downstream the TPR, interacts with the CS domain of Pih1p^15,25^. Taken together, these data suggest a model in which PIH-like and RPAP3-like proteins interact via an interface composed of a CS-domain on one side and a short region downstream the TPRs on the other. This type of interaction may also extend to DYX1C1 since its three isoforms, which differ in TPR domains and the downstream flanking region, interact with different PIH partners.

Our interaction assays indicate that cells may contain other related complexes. First, an R2T complex that contains RPAP3-iso-2 and RUVBL1/2 but lacks a PIH1-like component. This complex may be specialized in nuclear functions. Second, an R2SD complex composed of SPAG1, DNAAF2 and RUVBL1/2. It was not detected in our proteomic analyses and may thus form only in specific cell types. Finally, DYX1C1 isoforms associate with DNAAF2 and PIH1D3 but apparently without binding the RUVBLs. We do not exclude the possibility that additional proteins may be present, as the prefoldins in the case of R2TP. Overall, this study highlights the variety of R2TP-like complexes.

With the exception of PIH1D2, all the R2TP-related proteins analyzed here have been previously linked to the formation of cilia and to the assembly of axonemal dyneins^29,36,43^. Our and previous evolutionary analysis showed a loss of these proteins specifically in species lacking cilia (Fig. 1c and ^30^). However, several lines of evidence suggest that they have additional functions: (i) R2SP interacts and is involved in the biogenesis of proteins unrelated to cilia function; (ii) the strongest proteomic partner of DNAAF2 is RTF1, which is a nuclear protein involved in transcription; (iii) our CCDC103 two-hybrid screen performed with a testis library revealed only a few proteins related to cilia function (see Supplementary Data 2); (iv) GFP-tagged PIH1D3 and DYX1C1-iso-c are predominantly nuclear, with an accumulation in uncharacterized nuclear dots. In the future, it will be interesting to characterize the various functions of these R2TP-related proteins and to determine the balance of direct and indirect effects in cilia formation.

R2SP enrichment in testis could be due to two reasons: (i) it has testis-specific clients; (ii) it helps ubiquitous proteins to adapt the particular environment of testis. Our data suggest that both possibilities occur, since some putative clients of R2SP are enriched in testis, while others are ubiquitous. Indeed, some clients exhibited a stronger dependency on R2SP at 32 °C, the temperature of testis. This suggests that proteins selected to function at 37 °C may require additional help to function at 32 °C.

In the case of liprin-α2, we show that R2SP is required for its expression and association with its partners. Interestingly, the R2SP subunit SPAG1 binds several liprin-α2 partners while PIH1D2 binds liprin-α2 itself. This suggests a model in which the proteins to be assembled are brought together by R2SP, thus giving them the possibility to interact with each other and to access the chaperoning activity of the RUVBL1/2 ATPase (Fig. 7d). The independent recruitment of multiple subunits of client complexes may be a general mechanism of action for this class of chaperones.

Liprin-α2 is a conserved scaffolding protein expressed in the brain and to a lesser extends in testis (Gtex Portal,^35^). It plays an important role in neurons, where it participates to the organization of the synaptic active zone and in the coordinated exocytosis of pre-synaptic vesicles^44^. Interestingly, the acrosomal reaction also requires the simultaneous exocytosis of a large number of vesicles, and this process is also dependent on liprins^45^. A parallel has been thus drawn between the synapse and the acrosome, leading to the term acrosomal synapse^46^. Since PIH1D2 is also expressed in the brain, it will be interesting to determine whether R2SP participates to synaptic transmission, through its action on liprins.

## Methods

### Cell culture

HeLa Flp-In cells were a gift of S. Emiliani (Institut Cochin, Paris)^47^. HEK293 cells were from the ATCC collection. HeLa Flp-In and HEK293 cells were grown in Dulbecco’s modified eagle medium (DMEM) containing 10% fetal bovine serum (FBS), glutamin (2.9 mg/ml), and penicillin/streptomycin (10 U/ml), at 37 °C, 5% CO_2_. For SILAC, a 3×-FLAG-GFP tag was fused at the N-terminus of the indicated proteins and the fusions were stably expressed in HeLa H9 cells by Flp-In recombination, using the CMV promoter to drive expression^48^. Clones were selected in hygromycin (150 μg/ml), picked individually and characterized by Western blots and fluorescence microscopy.

### Plasmids and cloning

DNA cloning was performed using standard techniques and with the Gateway™ system (InVitrogen). For pairwise two-hybrid tests, plasmids were based on pACTII and pAS2ΔΔ ^48^. For the LUMIER assays, the baits and preys were expressed in HEK293 cells from the CMV promoter for the 3xFLAG-FFL fusions, or from the mouse L30 promoter for the RL fusions. The cDNAs were all of human origin except for RUVBL1 and RUVBL2, which were from mouse. Plasmids for in vitro expression in *Escherichia coli* are described below. Detailed maps and sequences are available upon request. RUVBL1, RUVBL2 are cloned in the pETDuet vector (Novagen) by manufacturer (GenScript) between *Nco*I and *Hind*III, and *Nde*I and *Xho*I, respectively. RPAP3-Cter PCR-amplified fragment were cloned between the *Nde*I and *Bam*H1 sites in custom pET-Based vector (pnEA-3CH, ^49^) (Supplementary Table 1).

### Purification of the human RUVBL1-RUVBL2

RUVBL1 carries an N-terminal 6×His-tag followed by thrombin cleavage site, while RUVBL2 has an N-terminal FLAG-tag followed by a TEV cleavage site. The RUVBL1-RUVBL2 complex was expressed in *Escherichia coli* (DE3) (Novagen, 71400), with 100 µM IPTG overnight at 18 °C. The complex was immobilized in a 5 ml Histrap ^TM^ HP (GE Healthcare) and eluted with 300 mM imidazole. Anti-Flag M2 Affinity Gel (Sigma) was used as a second affinity step. FLAG_FH8 tag was cleaved by incubating 18 h at 4 °C with 1% (w/w) HRV-3C protease (Thermo Fisher Scientific). Two size exclusion steps separated oligomeric species, FLAG_FH8 and protease. Superdex S200 and Superose 6 column (GE Healthcare) equilibrated in 20 mM Tris–HCl pH 8.0, 150 mM NaCl, 5% glycerol, 2 mM MgCl_2_ and 0.5 mM TCEP resulted in a stable dodecameric complex eluting as a single peak from Superose 6 (Supplementary Fig. 2C).

### Purification of the human RUVBL1-RUVBL2-RPAP3 complex

RUVBL1 and RUVBL2 were co-expressed *E. coli* (DE3; Novagen, 71400) containing the pRARE2 plasmid, during 24 h at 30 °C in EnPresso® B animal-free Media (BioSilta), by adding 100 µM of IPTG, in a New Brunswick™ (Innova®) 44R Shaker at 225 rpm. RUVBL1 was described previously^50^, while RUVBL2 carried a C-terminal FLAG_FH8 Tag^51^ preceded by a Human Rhino 3C cleavage site (HRV-3C). RPAP3_535-665_ did not any tag.

The RUVBL1-RUVBL2-RPAP3_535-665_ (R1R2R3) complex was purified, as described^50^, but in the presence of ADP and with replacement of the FlagTrap by a Hydrophobic interaction column (HIC), followed by the Superose 6 column. Peak fractions collected from the HisTrap were incubated with 5 mM CaCl_2_ during 1 h and loaded onto an HiPrep^TM^ Octyl FF 16/10 column (GE Healthcare) equilibrated in Buffer C (20 mM Tris–HCl pH 8.0, 200 mM NaCl, 5 % glycerol, 2 mM MgCl_2_, 5 mM CaCl_2_, 0.5 mM TCEP, 300 µM ADP). Bound proteins were eluted using Buffer D (Buffer C without CaCl_2_, supplemented with 5 mM EDTA). To remove the FLAG_FH8 tag the collected samples were incubated 18 h at 4 °C with 1% (w/w) HRV-3C protease (Thermo Fisher Scientific). To separate oligomeric species, we used a Superose 6 column equilibrated in 20 mM Tris–HCl pH 8.0, 150 mM NaCl, 5% glycerol, 2 mM MgCl_2_, 0.5 mM TCEP and 400 µM ADP. Elution resulted in a single peak containing a stable R1/R2 hexameric complex bound to RPAP3-Cter (Fig. 2f). The peak fractions were pooled and concentrated to 12.5 mg/ml using a 10 kDa Cut-off Amicon Ultra centrifugal filter (Millipore). All purification steps were carried out at room temperature and were monitored by NuPage Bis-Tris gels (Invitrogen, NP0302).

### Purification of RPAP3_535-665_ and NMR sample preparation

Recombinant ^13^C/^15^N-labeled RPAP3_535-665_ domain with a cleavable 6xHis-tag were overexpressed in *E. coli* (DE3) pRARE2 (Novagen) overnight at 20 °C in a minimal M9 medium complemented with ^15^N-NH_4_Cl and ^13^C-d6-Glucose and purified on TALON beads (Clonteth) in 25 mM HEPES, pH 7.5, 300 mM NaCl and eluted from the resin by cleavage with the HRV-3C protease (GE Healthcare). A final size exclusion chromatography (S75, GE Healthcare) performed in 10 mM NaPi, pH 6.4, 150 mM NaCl, and 0.5 mM TCEP provided ^15^N/^13^C-labeled samples at a concentration of 1 mM after concentration with Amicon Ultra-15 centrifugal filter unit (Millipore).

### NMR structure calculation

Classical 3D NMR spectra were recorded at 298 K on a 600 MHz AVANCE III spectrometer equipped with a cryoprobe. This permitted to obtain almost complete ^1^H, ^13^C and ^15^N resonance assignments of RPAP3_535-665_. Chemical shifts were referenced to DSS and derived into dihedral angle restraints with TALOS-N^52^. Automated procedure of CYANA 3.97^53^ was then used to derive distance restraints from 2D ^1^H-^1^H NOESY and 3D ^15^N- and ^13^C-NOESY-HSQC spectra, all recorded with a mixing time of 120 ms. The final sets of dihedral angle and inter-proton restraints were carefully checked and used to generate 100 CYANA structures, which were refined in explicit water using the AMBER-based Portal Server for NMR structures (AMPS-NMR;^54,55^). The 20 structures with the lowest constraint energies were selected as the most representative. In the final set of structures, 94.3% and 5.7% of the residues lie respectively in the most favored and allowed regions of the Ramachandran plot. All 3D structures were drawn with Pymol^56^.

### NMR interaction experiments

1D METHYL-SOFAST-HMQC spectra were recorded to monitor the binding of unlabeled RUVBL proteins to ^13^C-labeled RPAP3_535-665_. The ^1^H dimension was edited, which permitted to only detect ^1^H nuclei attached to ^13^C nuclei. Interaction experiments were performed at 298 K and at 600 MHz in the RUVBL1/2 buffer (20 mM Tris–HCl pH 8.0, 250 mM NaCl, 5% glycerol, 2 mM MgCl_2_, 0.5 mM TCEP). RUVBL1 was N-terminally tagged with a 6xHistidine and RUVBL2 with a 3xFLAG. Concentration of ^13^C-labeled proteins was around 10 µM. Protons attached to ^13^C nuclei lying between 5 and 35 ppm were selectively excited on a band width of 3 ppm and centered at 0 ppm. The relaxation delay was set to 150 ms and the number of scans to 2048. The final concentration ratio between unlabeled and labeled proteins was 1:1 (considering one monomer of RPAP3-Cter and one heterodimer of RUVBL1/2).

### Non-denaturing mass spectrometry analysis

For non-denaturing mass spectrometry analysis, samples were buffer exchanged against ammonium acetate (150 mM, pH 8.0) buffer (Sigma, St. Louis, MO, USA), using Zeba Spin desalting columns (Thermo Fisher Scientific, Rockford, IL, USA). Sample concentrations were determined by UV absorbance using a NanoDrop 2000 spectrophotometer (Thermo Fisher Scientific, France). Mass spectrometry analyses were carried out on a hybrid electrospray quadrupole time-of-flight mass spectrometer (Synapt G2 HDMS, Waters, Manchester, UK) coupled to an automated chip-based nanoelectrospray source (Triversa Nanomate, Advion Biosciences, Ithaca, U.S.A.) operating in the positive ion mode. Mass spectrometer calibration was performed using singly charged ions produced by a 2 mg/ml solution of cesium iodide in 2-propanol/water (1v/1v) over the m/z range 1000–20,000. Instrumental parameters have been optimized to get optimal high m/z ion transmission without dissociation of weak non-covalent interactions by raising the backing pressure to 6 mbar and the cone voltage to 100 V. Data interpretation was performed using MassLynx 4.1 (Waters, Manchester, UK).

### SPR interaction experiments

RPAP3-Cter protein was immobilized onto CM5 (Series S) sensor chips using standard amine coupling. HBS-N, which consisted of 10 mM HEPES, pH 7.4, 0.15 M NaCl, was used as the background buffer. The carboxymethyl surface of the chip was activated with 20 mM EDC and 5 mM NHS for 1.5 min. RPAP3_535-665_ was diluted in 10 mM Sodium Acetate, pH 5.5, to a concentration of 1 μg/ml. The protein was coupled to the surface with a 1 to 2 min injection time at a flow rate of 10 μl min^−1^. The remaining activated groups were blocked with a 5 min injection of 1.0 M ethanolamine, pH 8.5. Typically, 100 ± 10 response units (RU) were obtained for the immobilization of RPAP3-Cter protein. Negative controls were performed by immobilizing either Bovine Serum Albumin (BSA, Thermo Fisher Scientific) or human Cyclophilin D_43-207_ (CypD) with the same RU levels as RPAP3-Cter. CypD_43-207_ is a 22 kDa in-house purified protein with similar size to RPAP3-Cter and confirmed to be active through binding to Cyclosporin A. The RUVBL1-RUVBL2 complex was directly dissolved in running buffer (20 mM NaKPi pH 7.5, 150 mM NaCl, 5 mM MgCl2, 1 mM DTT, 0.05% P20). The RUVBL1-RUVBL2 complex was tested at 10 different concentrations using a 2-fold dilution series, with the highest concentration tested being 75.2 nM. Interaction analysis cycles consisted of a 300 s sample injection (30 µl min^−1^; association phase) followed by 600 s of buffer flow (dissociation phase). All sensorgrams were processed by first subtracting the binding response recorded from the control surface (reference spot), followed by subtracting of the buffer blank injection from the reaction spot. All datasets were fit to a simple 1:1 Langmuir interaction model (considering one molecule of RPAP3 binding one hexamer of RUVBL1/2), to determine the kinetic rate constants. Experiments were performed on a Biacore 4000 (Biacore AB, GE Healthcare Life Sciences, Uppsala, Sweden) at 25 °C and the interactions were evaluated using the provided Biacore 4000 evaluation software.

### SILAC proteomics

SILAC experiments were performed as previously described^7^. HeLa cells were grown for 15 days in each isotopically labeled media (CIL/Eurisotop), to ensure complete incorporation of isotopically labeled arginine and lysine (light label (K0R0, L) or semi-heavy label L-Lysine-^2^HCl (^2^H4, 96–98%)/L-Arginine-HCl (^13^C6, 99%) (K4R6, M) or L-Lysine-2HCl (^13^C6, 99%; ^15^N2, 99%)/L-Arginine-HCl (^13^C6, 99%; ^15^N4, 99%) heavy label (K8R10, H) (percentages represent the isotopic purity of the labeled amino acids). Six 15-cm diameter plates were used per SILAC condition. Cells were rinsed with PBS, trypsinized and cryogrinded in lysis buffer (0.5% triton X-100, 20 mM HEPES, pH 7.4, 150 mM NaCl, protease inhibitor cocktail). Extracts were incubated 20 min at 4 °C and clarified by centrifugation for 10 min at 20,000 g. For all IP experiments, extracts were pre-cleared by incubation with Protein G Sepharose beads (GE healthcare) for 1 h at 4 °C. The control was extracted from the SILAC light condition prepared from H9 HeLa cells that did not express the GFP fusion. Each extract was then incubated with 50 µl of GFP-Trap beads (gta-20, Chromotek) for 75 min at 4 °C, washed five times with lysis buffer, and beads from the different isotopic conditions were finally pooled. Bound proteins were eluted by adding 1% SDS to the beads and boiling for 10 min.

Reduction and alkylation were performed on the eluate with DTT (BDH 443553B, 10 mM) for 2 min at 95 °C followed by iodoacetamide treatment (Sigma I1149, 50 mM) for 30 min in the dark. Proteins were separated by SDS/PAGE and in gel-digested with trypsin in 20 mM NH_4_HCO_3_ (Trypsin Gold, Promega V5280). Ten slices were cut, and extracted peptides were resuspended in 0.1% formic acid/2% acetonitrile solution before being analyzed by mass spectrometry. Peptides were analyzed by nano-flow liquid chromatography coupled to Fourier transform tandem mass spectrometry (nanoLC-FT-MS/MS) using a LTQ Velos Pro Orbitrap Elite mass spectrometer coupled to an Ultimate 3000 (Thermo Fisher Scientific). Desalting and pre-concentration of samples were performed on-line on a Pepmap precolumn (0.3 mm 10 mm, Thermo Fisher Scientific) in buffer A (2% acetonitrile, 0.1% formic acid). A gradient consisting of 2–40% buffer B (B = 99.9% acetonitrile with 0.1% formic acid; 3–33 min) and 40–80% B (33–34 min) was used to separate peptides at 300 nL/min from a Pepmap capillary reversed-phase column (0.075 mm × 150 mm, Thermo Fisher Scientific). Mass spectra were acquired using a top-20 collision-induced dissociation (CID) data-dependent acquisition (DDA) method. The Orbitrap was programmed to perform a FT 400–1,400 Th mass scan (60,000 resolution) with the top 20 ions in intensity selected for collision-induced dissociation (CID) data-dependent acquisition (DDA) MS/MS in the LTQ. FT spectra were internally calibrated using a single lock mass (445.1200 Th). Target ion numbers were 500,000 for FT full scan on the Orbitrap and 10,000 MSn on the LTQ. Data were acquired using the Xcalibur software v2.2. Protein identification and quantitation were performed using the program MaxQuant (version 1.5.2.8; http://www.maxquant.org/). Few parameters were not default: database: human reference proteome set (canonical isoforms downloaded from Expasy on May 29th 2017); enzyme specificity trypsin/P; variable modifications: methionine oxidation and protein N-Acetylation; Fixed modifications: Cysteine carbamidomethylation; MS/MS tolerance: 0.5 Da; False Discovery Rate (FDR): 1%. In addition to the FDR, proteins were considered to be identified if they had at least two peptides including one unique/Razor peptide and they were considered quantified if they had at least one quantified SILAC pairs. Proteins labeled as REV (non-real proteins from the reverse database) and CONT (contaminants) were automatically discarded, as well as proteins that did not show any SILAC M/L, H/L and H/M ratio. B Significance calculation were done with the software Perseus v1.4.2, as previously described^57^ to highlight statistically significant protein ratios (*p* value < 0.05).

### LUMIER assays

HEK293 cells were seeded in 24-well plates and transfected with 450 ng of the RL fusion and 50 ng of the 3xFLAG-FFL fusion, with 1 μl of JetPrime (PolyPlus), as recommended by the manufacturer. 48 h later, cells were extracted for 15 min at 4 °C in 450 μl of HNTG containing protease inhibitor cocktail (Roche), and spun down at 4 °C and at 20,000 × *g* for 15 min. The IP was performed in duplicated, by putting 100 μl of the extract in each of four wells in a 96-well plate, with two wells being coated with anti-FLAG antibody (10 μg/ml in 1 × PBS; F1804 Sigma-Aldrich), and two control wells without antibodies. Plates were incubated for 3 h at 4 °C, and then washed 5 times with 300 μl of ice-cold HNTG, for 10 min at 4 °C for each wash. After the last wash, 10 μl of PBL buffer (Promega) was added in each well. To measure the input, 2 μl of extract and 8 μl of 1xPBL buffer was put in empty remaining wells. Plates were then incubated 5 min at room temperature, and FFL and RL luciferase activities were measured in IP and input wells, using the dual luciferase kit (Promega). Every transfection was performed at least twice as independent replicates. Co-IP efficiency was defined as the RL/FFL ratio in the pellet, divided by the RL/FFL ratio in the input. Unless otherwise stated, statistical significance was evaluated using Z-test assaying whether the co-IP efficiency in the anti-FLAG IP was more than 6 times higher than the mean values obtained in the control IP, done without antibodies. This threshold of 6 corresponds to the mean plus two standard deviations of the FLAG/control fold difference obtained with a set of 36 assays done with non-interacting proteins (see Supplementary Fig. 4A). It ensures that only specific interactions are identified. The image of Figs. 3c and 4c and Supplementary Figs. 2D, 4A, and 4B was created by the authors.

To coat the wells of the 96-well plates with M2 anti-FLAG antibodies, High-binding plates were used (Lumitrac, Greiner), and 70 μl of M2 antibody (10 μg/ml in 1× PBS; F1804 Sigma-Aldrich) was put in each well and incubated overnight at room temperature in the dark. The next day, wells were blocked with 300 μl of blocking buffer, for 1 hour at room temperature. IP control wells were treated the same way except that no antibody was put in the well. Blocking buffer was 3% BSA, 5% sucrose, 0.5% Tween 20, 1× PBS). HNTG buffer was 20 mM HEPES-KOH pH 7.9, 150 mM NaCl, 1% Triton X-100, 10% glycerol, 1 mM MgCl_2_, 1 mM EGTA. The values of LUMIER assays used in the bar plots are in Supplementary Data 3.

### Luciferase assays

H9 Hela cells were grown on 24-well plates and co-transfected with plasmids expressing a Flag-tagged Firefly luciferase (FFL) in fusion with the protein of interest, and with a plasmid coding Renilla luciferase as control (RL). After 48 h, cells were extracted in 100 µl of 1× PLB buffer (Promega) and incubated at 4 °C for 15 min. RL and FFL activities were measured on 96-well plates using 10 µl of cell extract and the dual-luciferase assay kit (Promega). Values obtained for FFL were normalized to RL values. Experiments were done at least in triplicate. The values used for the bar plot of Fig. 7a are in Supplementary Data 3.

### Microscopy

HeLa cells expressing the GFP-fusion of interest were plated on glass coverslips, fixed one day later, and mounted in Vectashield containing DAPI (Vector Laboratories). Cells were imaged using an upright epifluorescence microscope (Zeiss AxioImager Z1,) with a ×63 oil objective (NA 1.4). Images were captured with a sCMOS camera (Hamamatsu) and mounted in Photoshop.

### Genomes and Sequence analyses

Sequences were retrieved from the NCBI annotated databases (nr and EST, http://www.ncbi.nlm.nih.gov), using NCBI PHI-BLAST, as well as BLAST and Annotation search tools available in the Geneious 9.1.8 software package (Biomatters, http://www.geneious.com/). Amino acid sequences were aligned using MAFFT v7.017^58^. Orthology was determined by reciprocal BLAST analysis and domain architecture. The accession numbers used for Fig. 1c and Supplementary 3C are listed in Supplementary Data 4.

### Pairwise yeast two-hybrid assays

Plasmids pACT2 and pAS2ΔΔ were introduced into haploid *Saccharomyces cerevisiae* strains (Y187 and CG1945, respectively)^59^. Strains were crossed and diploids were selected on –Leu–Trp selective media and then plated on triple selective media (–Leu–Trp–His). Growth was assessed visually after 3 days at 30 °C. The strength of interactions was evaluated by comparing the number of clones growing on –Leu–Trp (selection of diploids) and –Leu–Trp–His plates (selection for interaction).

### Yeast two-hybrid screens

Yeast two-hybrid screening was performed by Hybrigenics Services, S.A.S., Paris, France (http://www.hybrigenics-services.com). The coding sequence for full-length (1–315) PIH1D2 (NCBI reference NM_138789) was PCR-amplified (Supplementary Table 1) and cloned into pB27 plasmid, which derives from pBTM116^60^. PIH1D1 was fused at the C-terminal end of LexA (LexA-PIH1D2). Bait sequence integrity was checked by sequencing. Two random-primed cDNA library were constructed in derivative of the P6 plasmid pGADGH^61^, and were used for the screens. The first was made from human testis cDNAs, and the second one with cDNAs made from three human lung cancer cell lines: A549, H1703, and H460.

As many as 31 and 51 million clones were screened for the human testis and the human lung cancer library, respectively. This represents 3-fold and 5-fold the complexity of the respective libraries. A mating strategy was used for the screens, using on one side L40ΔGal4 (Mata) yeast strains transformed with the bait, and on the other side the strain YHGX13 (Y187 ade2-101::loxP-kanMX-loxP, Matα) containing the library, as previously described^59^. For the testis, screen, 182 His+colonies were selected on a medium lacking tryptophan, leucine and histidine, while for the lung cancer library, 345 His+colonies were selected on a medium lacking tryptophan, leucine and histidine and supplemented with 50 mM 3-aminotriazole to retain an optimal selectivity. Between 2 and 10 million diploid were tested. The 5′ and 3′ junctions of the prey insert were analyzed by capillary sequencing after yeast lysis and PCR amplification. The resulting sequences were compared to Human GenBank (NCBI) for prey identification.

A statistical analysis of the results has been conduct to define a confidence score (PBS, for Predicted Biological Score), as previously described^62^. First, the number of independent prey, the localization of the prey fusion, and the reading frames were taken into account to define the local score. Second, a global connectivity study using all the screens conducted at Hybrigenics using the same libraries was applied. This second analysis identify False positive (Score F), and highly connected preys (score E). The other score defines a probability for an interaction to be identify by chance, and are divided in four categories, from A (highest confidence) to D (lowest confidence). The PBS scores have been shown to positively correlate with the biological significance of interactions^63,64^.

### Quantification and statistical analysis

Statistical tests were done with Excel.

### Data availability

Data supporting the findings of this manuscript are available from the corresponding authors upon reasonable request. 3D coordinates and NMR chemical shifts of RPAP3_535-665_ were deposited in the Protein Data Bank and in the Biological Magnetic Resonance Data Bank under respective entry codes 6EZ4 and 34200. The SILAC data are accessible in the ProteomeXchange Database with the following accession numbers: RPAP3-Cter and mutants (PXD009518), PIH1D2 (PXD009520), PIH1D3 (PXD009499), DNAAF2 (PXD009501), CCDC103 (PXD009498).

## Electronic supplementary material

Supplementary Information

Description of Additional Supplementary Files

Supplementary Data 1

Supplementary Data 2

Supplementary Data 3

Supplementary Data 4

## References

[1] Zhao R (2005). Navigating the chaperone network: an integrative map of physical and genetic interactions mediated by the hsp90 chaperone. Cell.

[2] Houry WA, Bertrand E, Coulombe B (2018). The PAQosome, an R2TP-based chaperone for quaternary structure formation. Trends Biochem. Sci..

[3] Boulon S (2008). The Hsp90 chaperone controls the biogenesis of L7Ae RNPs through conserved machinery. J. Cell Biol..

[4] Bizarro J (2015). NUFIP and the HSP90/R2TP chaperone bind the SMN complex and facilitate assembly of U4-specific proteins. Nucleic Acids Res..

[5] Cloutier P (2009). High-resolution mapping of the protein interaction network for the human transcription machinery and affinity purification of RNA polymerase II-associated complexes. Methods.

[6] Malinova A (2017). Assembly of the U5 snRNP component PRPF8 is controlled by the HSP90/R2TP chaperones. J. Cell Biol..

[7] Boulon S (2010). HSP90 and its R2TP/Prefoldin-like cochaperone are involved in the cytoplasmic assembly of RNA polymerase II. Mol. Cell.

[8] Cloutier P, Coulombe B (2010). New insights into the biogenesis of nuclear RNA polymerases?. Biochem. Cell Biol..

[9] Jeronimo C (2007). Systematic analysis of the protein interaction network for the human transcription machinery reveals the identity of the 7SK capping enzyme. Mol. Cell.

[10] Horejsi Z (2010). CK2 phospho-dependent binding of R2TP complex to TEL2 is essential for mTOR and SMG1 stability. Mol. Cell.

[11] Takai H, Xie Y, de Lange T, Pavletich NP (2010). Tel2 structure and function in the Hsp90-dependent maturation of mTOR and ATR complexes. Genes Dev..

[12] Boulon S, Bertrand E, Pradet-Balade B (2012). HSP90 and the R2TP co-chaperone complex: building multi-protein machineries essential for cell growth and gene expression. RNA Biol..

[13] von Morgen P (2017). MRE11 stability is regulated by CK2-dependent interaction with R2TP complex. Oncogene.

[14] Horejsi Z (2014). Phosphorylation-dependent PIH1D1 interactions define substrate specificity of the R2TP cochaperone complex. Cell Rep..

[15] Pal M. et al. Structural basis for phosphorylation-dependent recruitment of Tel2 to Hsp90 by Pih1. *Structure* **22**, 805–818 (2014). 10.1016/j.str.2014.04.001 PMC4058522 24794838

[16] Zhou CY (2017). Regulation of Rvb1/Rvb2 by a domain within the INO80 chromatin remodeling complex implicates the yeast rvbs as protein assembly chaperones. Cell Rep..

[17] Bizarro J (2014). Proteomic and 3D structure analyses highlight the C/D box snoRNP assembly mechanism and its control. J. Cell Biol..

[18] Matias PM, Gorynia S, Donner P, Carrondo MA (2006). Crystal structure of the human AAA+protein RuvBL1. J. Biol. Chem..

[19] Ewens CA (2016). Architecture and nucleotide-dependent conformational changes of the Rvb1-Rvb2 AAA+complex revealed by cryoelectron microscopy. Structure.

[20] Lakomek K, Stoehr G, Tosi A, Schmailzl M, Hopfner KP (2015). Structural basis for dodecameric assembly states and conformational plasticity of the full-length AAA+ATPases Rvb1. Rvb2. Structure.

[21] Cloutier P (2017). R2TP/Prefoldin-like component RUVBL1/RUVBL2 directly interacts with ZNHIT2 to regulate assembly of U5 small nuclear ribonucleoprotein. Nat. Commun..

[22] McKeegan KS, Debieux CM, Watkins NJ (2009). Evidence that the AAA+proteins TIP48 and TIP49 bridge interactions between 15.5K and the related NOP56 and NOP58 proteins during box C/D snoRNP biogenesis. Mol. Cell Biol..

[23] Machado-Pinilla R, Liger D, Leulliot N, Meier UT (2012). Mechanism of the AAA+ATPases pontin and reptin in the biogenesis of H/ACA RNPs. RNA.

[24] Quinternet M (2015). Structure/function analysis of protein-protein interactions developed by the yeast Pih1 platform protein and its partners in box C/D snoRNP assembly. J. Mol. Biol..

[25] Back R (2013). High-resolution structural analysis shows how Tah1 tethers Hsp90 to the R2TP complex. Structure.

[26] Rivera-Calzada A. et al. The structure of the R2TP complex defines a platform for recruiting diverse client proteins to the HSP90 molecular chaperone system. *Structure* **25**, 1145–1152 (2017). 10.1016/j.str.2017.05.016 PMC5501727 28648606

[27] Tian S (2017). Pih1p-Tah1p Puts a lid on hexameric AAA+ATPases Rvb1/2p. Structure.

[28] Paci A (2012). The stability of the small nucleolar ribonucleoprotein (snoRNP) assembly protein Pih1 in *Saccharomyces cerevisiae* is modulated by its C terminus. J. Biol. Chem..

[29] Tarkar A (2013). DYX1C1 is required for axonemal dynein assembly and ciliary motility. Nat. Genet..

[30] Yamamoto R, Hirono M, Kamiya R (2010). Discrete PIH proteins function in the cytoplasmic preassembly of different subsets of axonemal dyneins. J. Cell. Biol..

[31] Quinternet M, Starck JP, Delsuc MA, Kieffer B (2012). Unraveling complex small-molecule binding mechanisms by using simple NMR spectroscopy. Chemistry.

[32] Wendler P, Ciniawsky S, Kock M, Kube S (2012). Structure and function of the AAA+nucleotide binding pocket. Biochim. Biophys. Acta.

[33] Yoshida M (2013). RPAP3 splicing variant isoform 1 interacts with PIH1D1 to compose R2TP complex for cell survival. Biochem. Biophys. Res. Commun..

[34] Schopf FH, Biebl MM, Buchner J (2017). The HSP90 chaperone machinery. Nat. Rev. Mol. Cell Biol..

[35] Thul PJ (2017). A subcellular map of the human proteome. Science.

[36] Olcese C (2017). X-linked primary ciliary dyskinesia due to mutations in the cytoplasmic axonemal dynein assembly factor PIH1D3. Nat. Commun..

[37] Stryker E, Johnson KGLAR (2007). liprin alpha and the regulation of active zone morphogenesis. J. Cell. Sci..

[38] Chia PH, Patel MR, Wagner OI, Klopfenstein DR, Shen K (2013). Intramolecular regulation of presynaptic scaffold protein SYD-2/liprin-alpha. Mol. Cell. Neurosci..

[39] Wei Z (2011). Liprin-mediated large signaling complex organization revealed by the liprin-alpha/CASK and liprin-alpha/liprin-beta complex structures. Mol. Cell.

[40] Zhao R, Houry WA (2005). Hsp90: a chaperone for protein folding and gene regulation. Biochem. Cell. Biol..

[41] Benbahouche Nel H (2014). Drosophila Spag is the homolog of RNA polymerase II-associated protein 3 (RPAP3) and recruits the heat shock proteins 70 and 90 (Hsp70 and Hsp90) during the assembly of cellular machineries. J. Biol. Chem..

[42] Roe SM (2004). The Mechanism of Hsp90 regulation by the protein kinase-specific cochaperonep50(cdc37). Cell.

[43] Omran H (2008). Ktu/PF13 is required for cytoplasmic pre-assembly of axonemal dyneins. Nature.

[44] Torres VI, Inestrosa NC (2017). Vertebrate presynaptic active zone assembly: a role accomplished by diverse molecular and cellular mechanisms. Mol. Neurobiol..

[45] Joshi CS, Khan SA, Khole VV (2014). Regulation of acrosome reaction by Liprinalpha3, LAR and its ligands in mouse spermatozoa. Andrology.

[46] Joshi CS, Suryawanshi AR, Khan SA, Balasinor NH, Khole VV (2013). Liprinalpha3: a putative estrogen regulated acrosomal protein. Histochem. Cell. Biol..

[47] Tantale K. et al. A single-molecule view of transcription reveals convoys of RNA polymerases and multi-scale bursting. *Nat. Commun.* **7**, 12248 (2016). 10.1038/ncomms12248 PMC4974459 27461529

[48] Hallais M (2013). CBC-ARS2 stimulates 3′-end maturation of multiple RNA families and favors cap-proximal processing. Nat. Struct. Mol. Biol..

[49] Diebold M. L., Fribourg S., Koch M., Metzger T., Romier C. Deciphering correct strategies for multiprotein complex assembly by co-expression: application to complexes as large as the histone octamer. *J. Struct. Biol*. **175**, 178–188 (2011). 10.1016/j.jsb.2011.02.001 21320604

[50] Gorynia S, Matias PM, Bandeiras TM, Donner P, Carrondo MA (2008). Cloning, expression, purification, crystallization and preliminary X-ray analysis of the human RuvBL1-RuvBL2 complex. Acta Crystallogr..

[51] Costa SJ (2013). The Fh8 tag: a fusion partner for simple and cost-effective protein purification in *Escherichia coli*. Protein Expr. Purif..

[52] Shen Y, Bax A (2013). Protein backbone and sidechain torsion angles predicted from NMR chemical shifts using artificial neural networks. J. Biomol. NMR.

[53] Lopez-Mendez B, Guntert P (2006). Automated protein structure determination from NMR spectra. J. Am. Chem. Soc..

[54] Bertini I, Case DA, Ferella L, Giachetti A, Rosato A (2011). A Grid-enabled web portal for NMR structure refinement with AMBER. Bioinformatics.

[55] Casey JP (2015). A case report of primary ciliary dyskinesia, laterality defects and developmental delay caused by the co-existence of a single gene and chromosome disorder. BMC Med. Genet..

[56] The PyMOL Molecular Graphics System, V.1.8 (Schrödinger Inc., 2015).

[57] Cox J (2014). Accurate proteome-wide label-free quantification by delayed normalization and maximal peptide ratio extraction, termed MaxLFQ. Mol. Cell. Proteom..

[58] Katoh K, Misawa K, Kuma K, Miyata T (2002). MAFFT: a novel method for rapid multiple sequence alignment based on fast Fourier transform. Nucleic Acids Res..

[59] Fromont-Racine M, Rain JC, Legrain P (1997). Toward a functional analysis of the yeast genome through exhaustive two-hybrid screens. Nat. Genet..

[60] Vojtek AB, Hollenberg SM (1995). Ras–Raf interaction: two-hybrid analysis. Methods Enzymol..

[61] Bartel P, Chien CT, Sternglanz R, Fields S (1993). Elimination of false positives that arise in using the two-hybrid system. Biotechniques.

[62] Formstecher E (2005). Protein interaction mapping: a Drosophila case study. Genome Res..

[63] Rain JC (2001). The protein-protein interaction map of Helicobacter pylori. Nature.

[64] Wojcik J, Boneca IG, Legrain P (2002). Prediction, assessment and validation of protein interaction maps in bacteria. J. Mol. Biol..

