# Supplementary material for: The RPAP3-Cterminal domain identifies R2TP-like quaternary chaperones
Source: Nat Commun. 2018 May 29;9:2093. doi: 10.1038/s41467-018-04431-1 (PMC5974087; doi:10.1038/s41467-018-04431-1)
Supplement: Supplementary file 1 — Supplementary Information [file 41467_2018_4431_MOESM1_ESM.pdf]

Supplemental Figures and Table for

The RPAP3-Cterminal domain identifies R2TP-like quaternary chaperones

Chloé Maurizy *et al.*

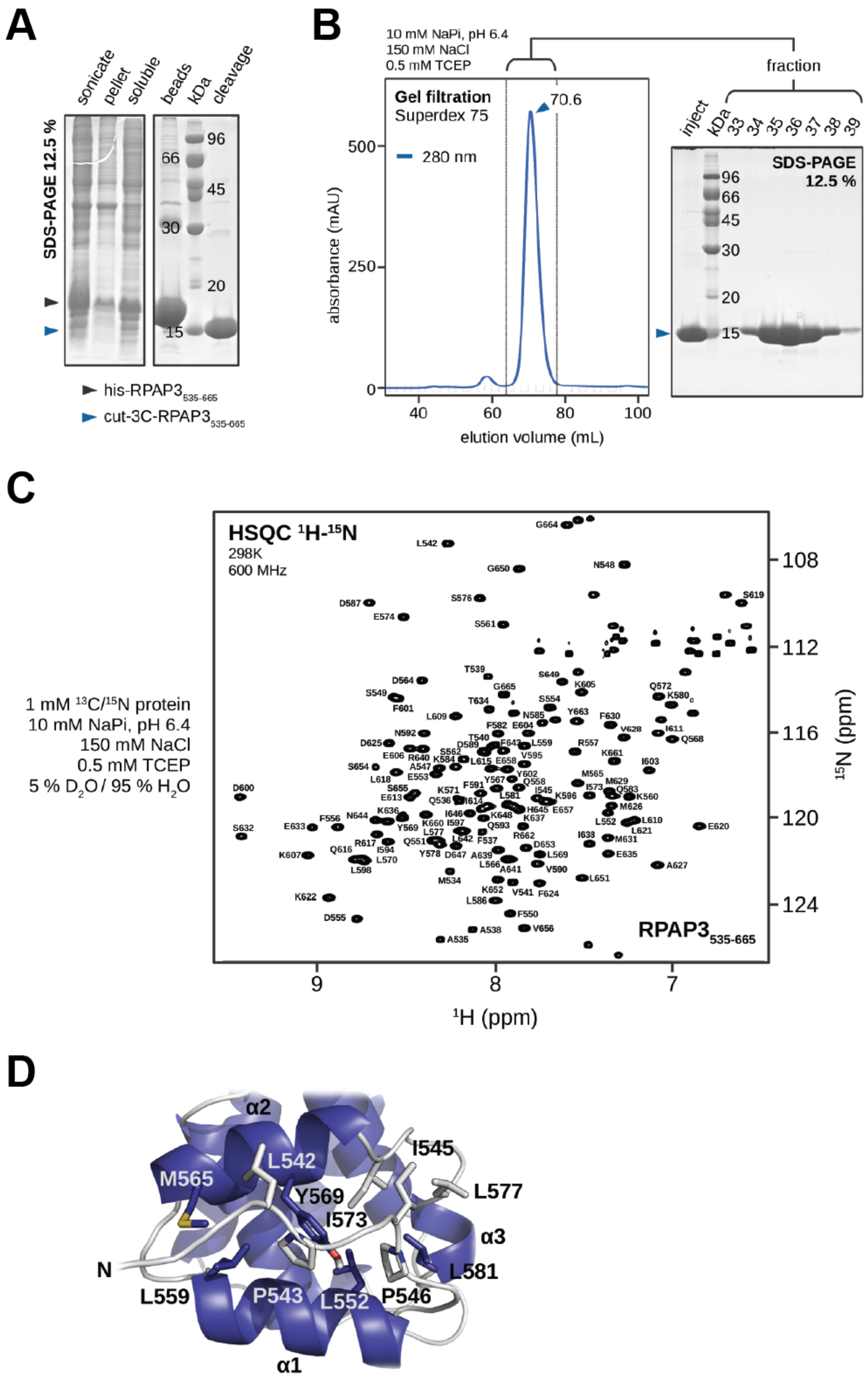

Supplementary Figure 1: Purification of RPAP3-Cter.

A-Purification of bacterially produced RPAP3-Cter. Left panel: Coomassie-stained gel loaded with extracts of bacteria overexpressing His-tagged RPAP3-Cter (fragment 535-665 of human RPAP3 isoform 1). Right panel: beads, molecular weight marker, and eluate of the beads digested with 3C protease. Arrows indicate the size of the tagged and untagged RPAP3-Cter.

B-Gel filtration profile of RPAP3-Cter eluted from beads (left), and Coomassie-stained gels of the various gel-filtration fractions (right).

C-HSQC ^{1}H-^{15}N NMR spectrum of RPAP3-Cter, with the assignment of the signals.

D-Highlights of the interaction of the N-terminal loop that folds back on the helix bundle (ribbon representation). The side chains of the residues involved are labeled.

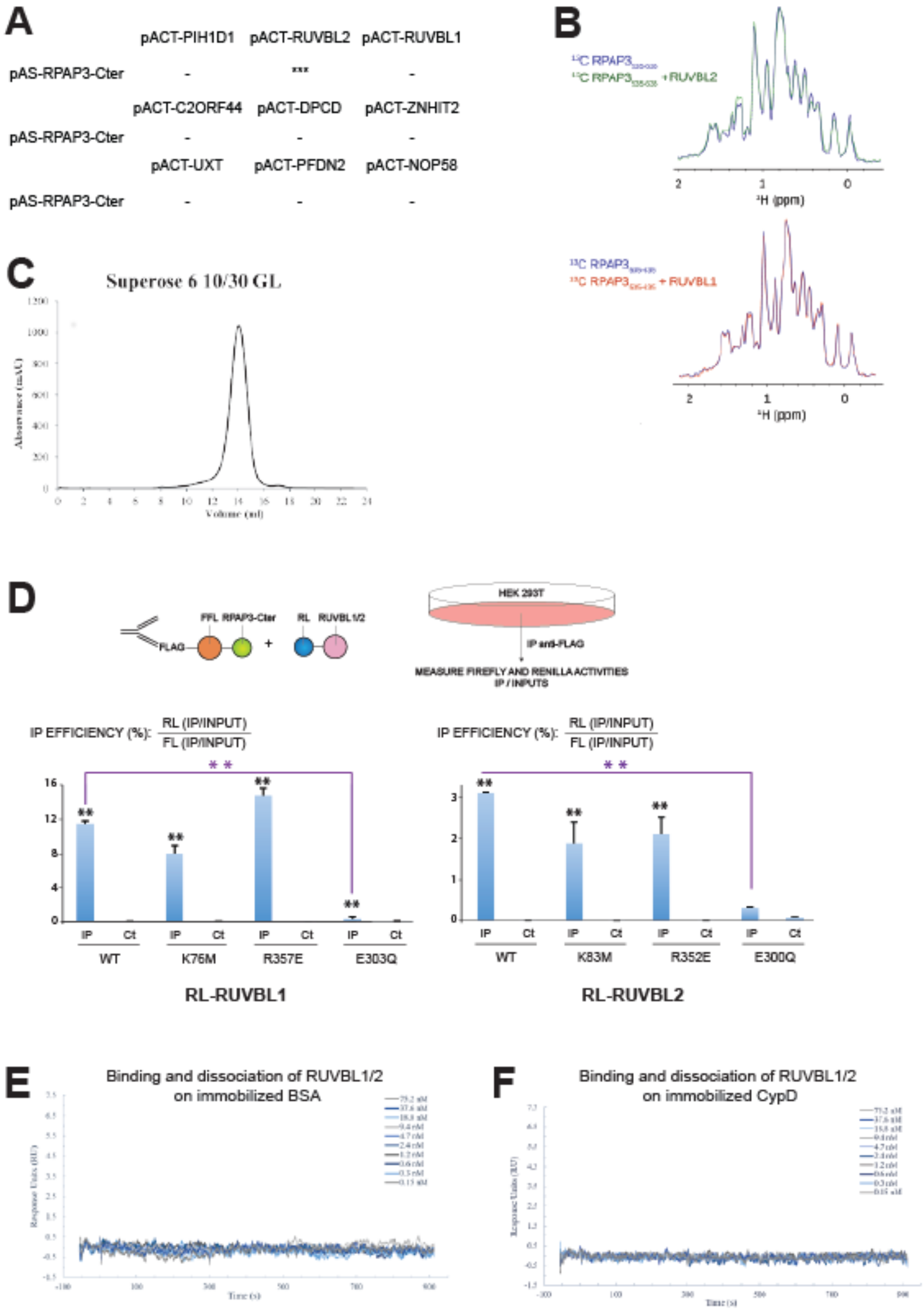

Supplementary Figure 2: RPAP3-Cter directly binds RUBL1/2 hexamers.

A-Yeast two-hybrid analysis of putative RPAP3-Cter partners. Yeast strains expressing a Gal4-DBD-RPAP3-Cter fusion (pAS-RPAP3-Cter) were crossed with strains expressing the indicated proteins fused to Gal4 activation domain (pACT). "***" denotes a positive interaction (growth on selective media), while "–" denotes an absence of interaction.

B-1D METHYL-SOFAST-HMQC NMR spectra in the methyl region of 13C-labeled RPAP3-Cter alone (blue line), or mixed with recombinant RUVBL1 (red line, top panel), or RUVBL2 (green line, bottom panel). Legend as in Figure 2C.

C-*In vitro* SPR binding assays of BSA (Bovine Serum Albumine) with RUVBL1/2. The graph depicts the response upon RUVBL1/2 injection (t=0s), or upon washing (t=300s), on immobilized BSA surfaces. X-axis: time (s); y-axis: response (arbitrary units).

D-LUMIER assay showing the in vivo interaction between RPAP3-Cter and RUVBL proteins. Legend as in 3C. **: p-value<0.001 (Z-test).

E-In vitro SPR binding assays of BSA with RUVBL1/2, without added nucleotide. The graph depicts the response of RUVBL1/2 injection (t=0s), or washing (t=300s), on immobilized BSA molecules. X-axis: time (s); y-axis: response (arbitrary units).

F-Legend as in E except that human His-tagged Cyclophilin D (CYPD) was immobilized on the surface of chip.

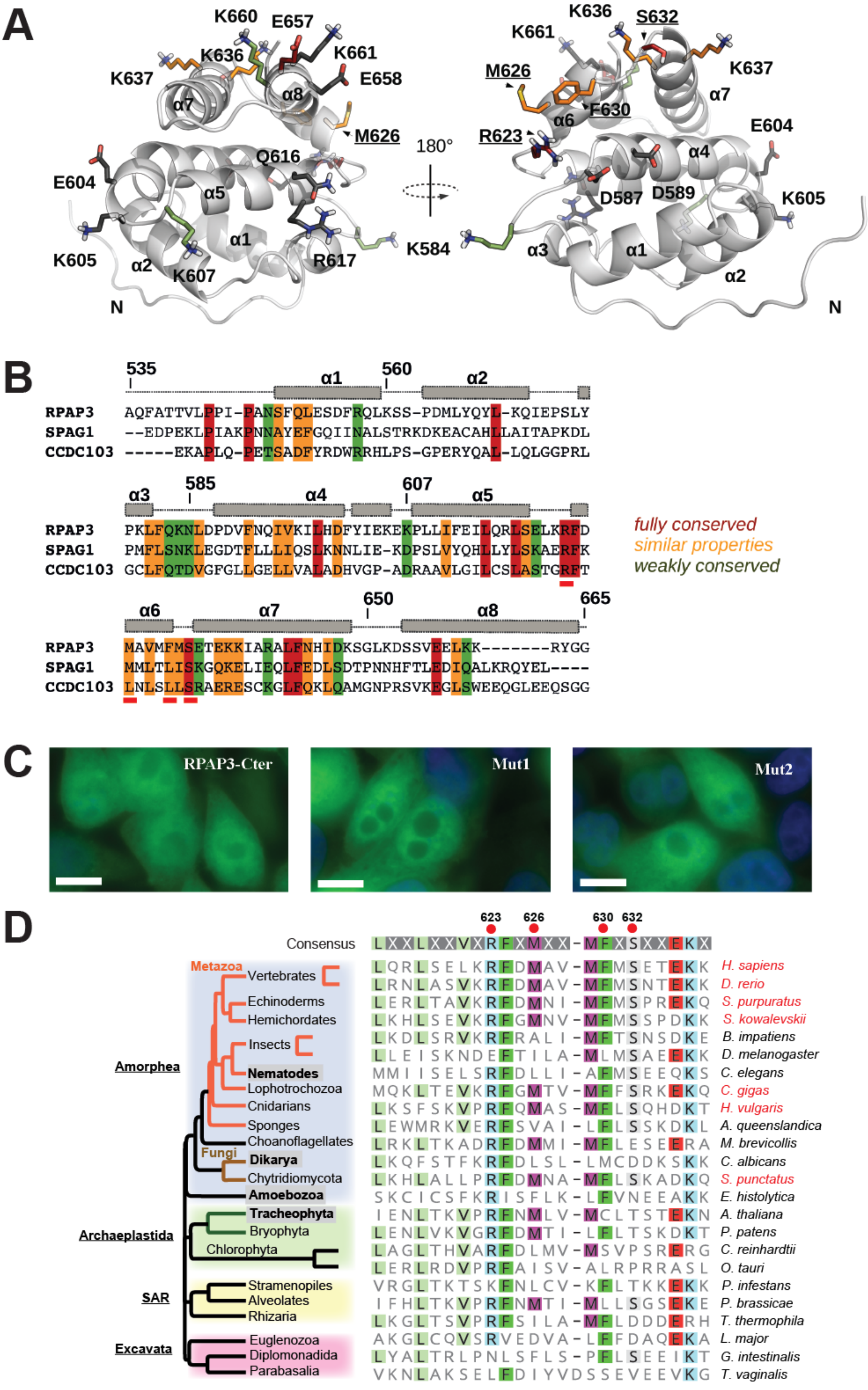
A
K660
E657
K636
K661
K637
E658
α7
α8
M626
E604
Q616
α5
α1
K605
α2
K607
R617
K584
N
180°
K636
S632
K661
M626
K637
F630
α7
α6
R623
E604
α4
D587
D589
K605
α3
α1
α2
N
B
535
α1
560
α2
RPAP3 AQFATTVLPPI-PANSFQLESDFRQLKSS-PDMLYQYL-KQIEPSLY
SPAG1 --EDPEKLPIAKPNNAYEFGQIINALSTRKDKEACAHLLAITAPKDL
CCDC103 -----EKAPLQ-PETSADFYRDWRRHLPS-GPERYQAL-LQLGGPRL
α3
585
α4
607
α5
RPAP3 PKLFQKNLDPDVFNQIVKILHDFYIEKEKPLLIFEILQRLSELKRFD
SPAG1 PMFLSNKLEGDTFLLLIQSLKNNLIE-KDPSLVYQHLLYLSKAERFK
CCDC103 GCLFQTDVGFGLLGELLVALADHVGP-ADRAAVLGILCSLASTGRFT
fully conserved
similar properties
weakly conserved
α6
α7
650
α8
665
RPAP3 MAVMFMSETEKKIARALFNHIDKSGLKDSSVEELKK-------RYGG
SPAG1 MMLTLISKGQKELIEQLFEDLSDTPNNHFTLEDIQALKRQYEL----
CCDC103 LNLSLLSRAERESCKGLFQKLQAMGNPRSVKEGLSWEEQGLEEQSGG
C
RPAP3-Cter
Mut1
Mut2
D
623
626
630
632
Consensus
Metazoa
Vertebrates
Echinoderms
Hemichordates
Insects
Nematodes
Lophotrochozoa
Cnidarians
Sponges
Choanoflagellates
Fungi
Dikarya
Chytridiomycota
Amoebozoa
Tracheophyta
Bryophyta
Chlorophyta
Stramenopiles
Alveolates
Rhizaria
Euglenozoa
Diplomonadida
Parabasalia
Amorphea
Archaeplastida
SAR
Excavata
H. sapiens
D. rerio
S. purpuratus
S. kowalevskii
B. impatiens
D. melanogaster
C. elegans
C. gigas
H. vulgaris
A. queenslandica
M. brevicollis
C. albicans
S. punctatus
E. histolytica
A. thaliana
P. patens
C. reinhardtii
O. tauri
P. infestans
P. brassicae
T. thermophila
L. major
G. intestinalis
T. vaginalis

Supplementary Figure 3. Characterization of RPAP3-Cter mutants that do not bind RUVBL1/2.

A-3D structure of RPAP3-Cter, with the number and position of the mutated surface residues.

B-Alignment of RPAP3-Cter with the two other human proteins bearing a similar domain (SPAG1 and CCDC103). Underlined in red: the RPAP3-Cter amino-acids required for the interaction with RUVBL1/2.

C-Intracellular localization of wild-type and mutant RPAP3-Cter protein. Panels are micrographs of HeLa cells expressing the indicated protein fused to GFP. Green: GFP fluorescence; blue: DAPI signals. Scale bar: 10 microns.

D- Conservation of the residues of RPAP3-Cter critical for RUVBL1/2 binding.

RPAP3 sequences from the indicated species were aligned and the portion corresponding to the human 615-637 amino acids are shown. The residues critical to RUVBL1/2 binding, i.e. R623, M626, F630 and S632 are figured by a red dot. Species in red indicate full conservation of the four amino acids.

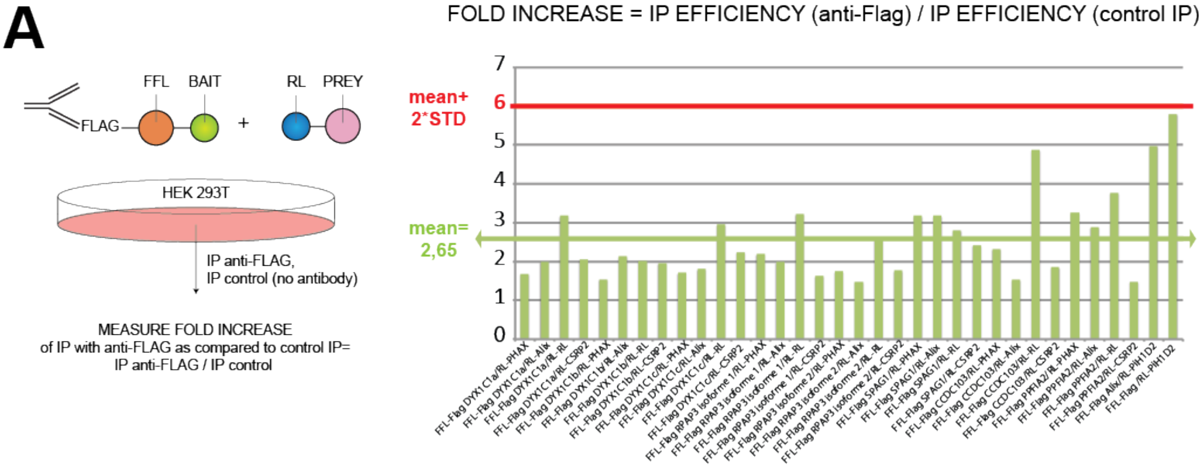

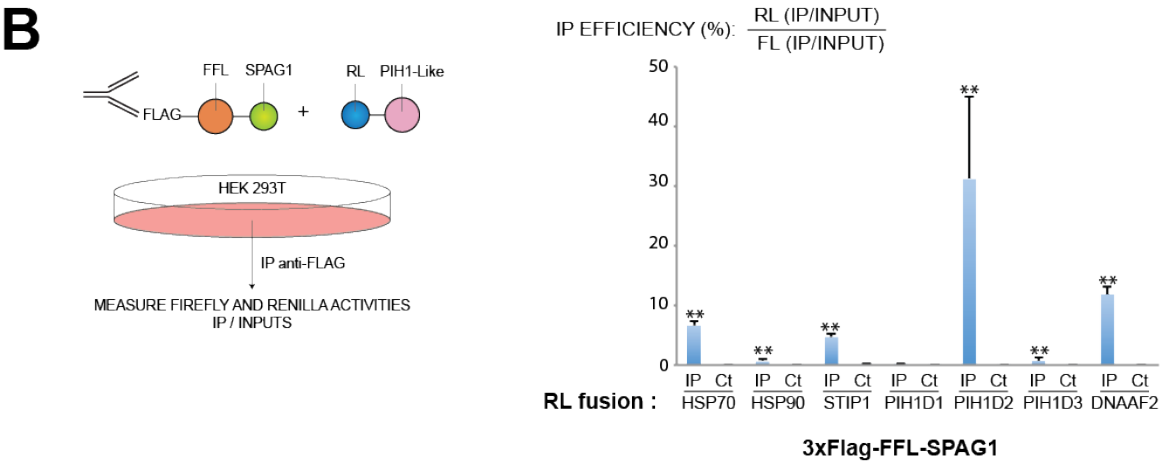

C

Fold enrichment over control (pValue)

	DYX1C1-iso-a	DYX1C1-iso-b	DYX1C1-iso-c	RPAP3-iso-1	RPAP3-iso-2	SPAG1	CCDC103
HSP70	34 (3.10^{-3})	9 (0.2)	49 (4.10^{-27})	114 (9.10^{-6})	70 (2.10^{-2})	223 (2.10^{-81})	37 (2.10^{-2})
HSP90	58 (1.10^{-150})	18 (3.10^{-39})	51 (6.10^{-3})	61 (2.10^{-11})	29 (5.10^{-4})	103 (1.10^{-3})	42 (1.10^{-25})
STIP1	164 (4.10^{-6})	94 (2.10^{-3})	146 (5.10^{-14})	249 (3.10^{-3})	131 (5.10^{-3})	212 (6.10^{-85})	196 (2.10^{-2})
PIH1D1	2 (1)	2 (1)	5 (1)	2232 (7.10^{-4})	26 (3.10^{-3})	7 (0.5)	3 (1)
PIH1D2	2 (1)	2 (1)	5 (0.8)	5 (0.8)	12 (4.10^{-2})	935 (3.10^{-6})	3 (1)
PIH1D3	45 (1.10^{-44})	10 (2.10^{-4})	78 (3.10^{-3})	13 (2.10^{-8})	10 (6.10^{-2})	12 (5.10^{-3})	9 (7.10-2)
DNAAF2	2910 (1.10^{-4})	49 (2.10^{-10})	18 (5.10^{-4})	38 (5.10^{-10})	96 (3.10^{-11})	651 (9.10^{-4})	10 (0.1)

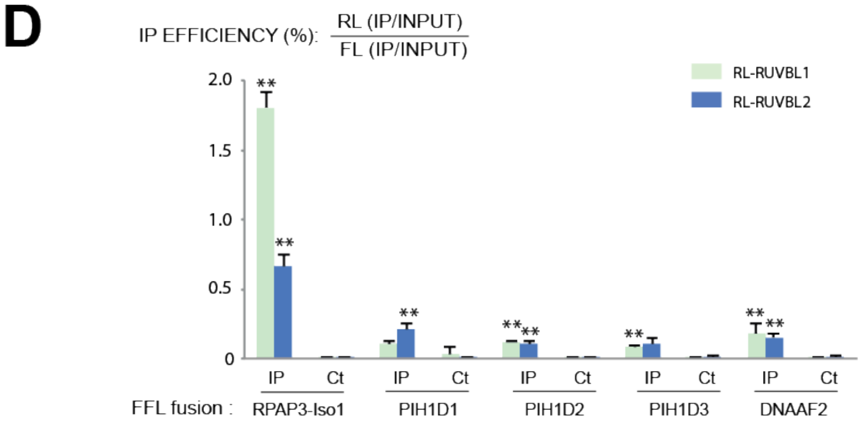

Supplementary Figure 4. Interaction of RPAP3-like and PIH-like proteins.

A-Control LUMIER assay with Alix, FFL, PHAX and CSRP2. The graph depicts fold enrichment of the FLAG IP vs the control IP (RL[IP FLAG] / RL[IP Ct]), for a series of control assays.

B-LUMIER interaction assay of SPAG1 with chaperones and PIH-like proteins. Legend as in Figure 3C.

C-Summary of pairwise LUMIER interaction assays performed with the indicated proteins. The values are fold enrichment of a FLAG IP over a control IP done in absence of antibodies (RL[IP FLAG] / RL[IP Ct]). In parenthesis: pValues calculated with Z-test assaying whether the RL signal in the anti-FLAG IP is more than 6 times higher than in mean of the corresponding control IP done without antibodies.

D-LUMIER interaction assays of PIH-like proteins with RUVBL1 and RUVBL2. Legend as in Figure 3C. **: p-value<0.001 (Z-test).

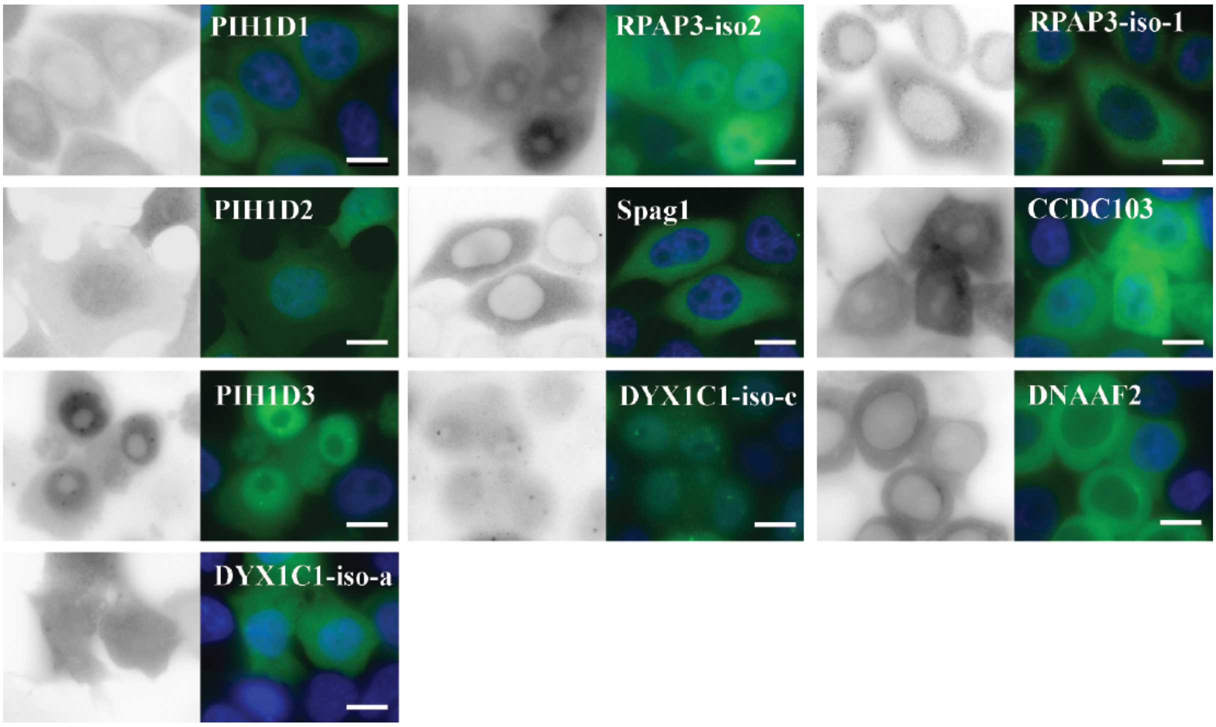

Supplementary Figure 5: Localization of RPAP3-like and PIH-like proteins.

Panels are micrographs of HeLa cells expressing the indicated protein fused to GFP. Green and black (left panels): GFP fluorescence; blue: DAPI signals. Scale bar: 10 microns.

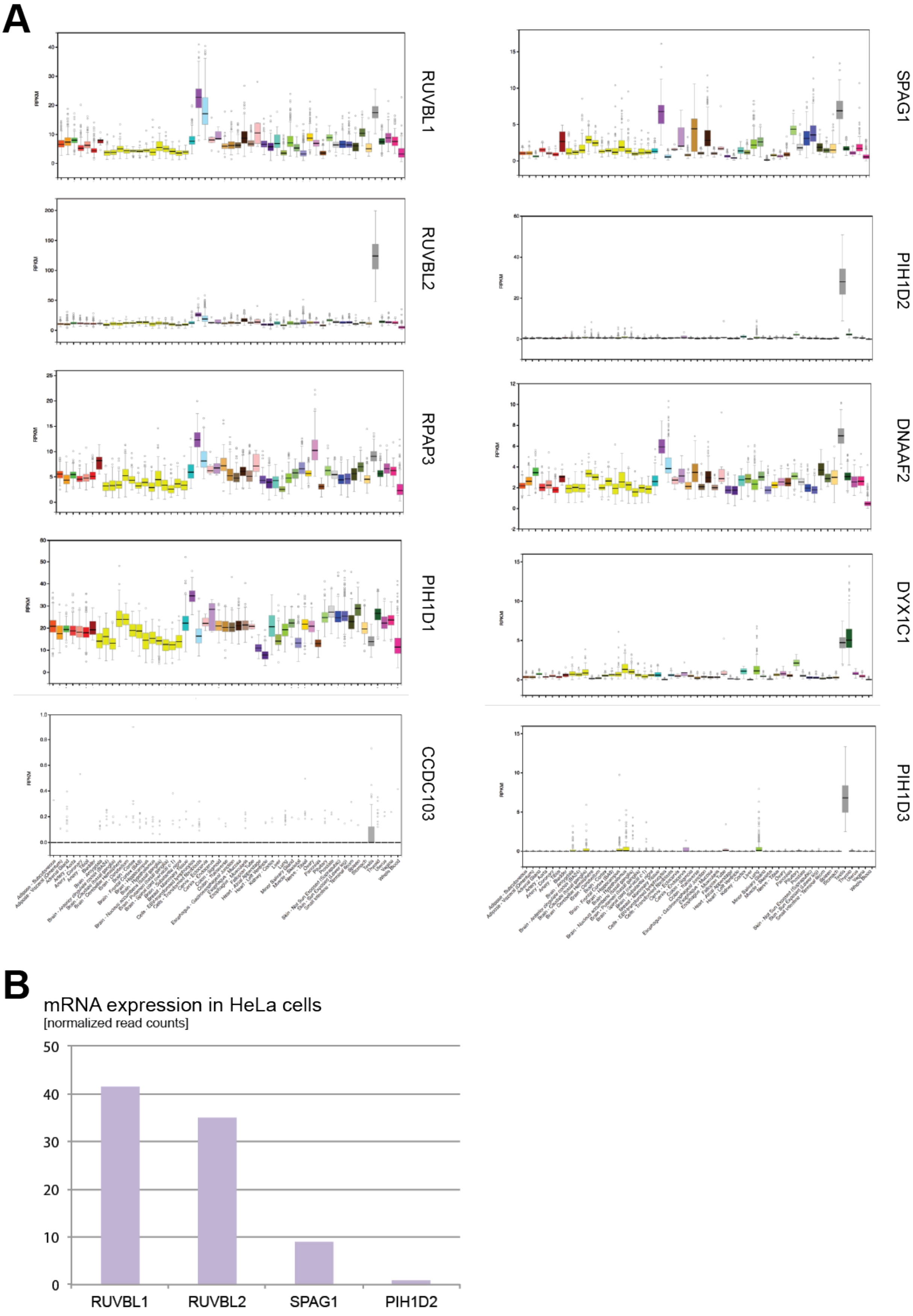

Supplementary Figure 6: Tissue-specific expression of R2TP, RPAP3-like and PIH-like proteins.

A-Graphs displaying the mRNA expression levels (RPKM) of the RPAP3-like and PIH1-like proteins in various human organs. Data are from GTex portal (https://gtexportal.org/home/).
B-Graph displaying the expression levels of mRNAs encoding R2SP proteins in HeLa cells (normalized read counts).

Supplementary Table 1: primer sequences

	**RPAP3-Cter** (NMR)
Oligonucleotide forward	GGATATCCATATGGCTCAGTTTGCCACAACTGTT
Oligonucleotide reverse	GGGGGATCCTTAACCACCGTATCTTTTCTTGAGTTC
	**RPAP3-Cter** (Gateway)
Oligonucleotide forward	GGG ACA AGT TTG TAC AAA AAA GCA GGC T CCATGCAGTTTGCCACAACTGTTCTTC
Oligonucleotide reverse	GGGG GAC CAC TTT GTA CAA GAA AGC TGG GTT TTA ACCACCGTATCTTTTCTTGAG
	**RPAP3-Cter mut1** (R623A-M626A) (IP SILAC)
Oligonucleotide forward	GACTTTCTGAACTAAAA GCG TTTGAT GCG GCAGTGATGTTTATG
Oligonucleotide reverse	CATAAACATCACTGCCGCATCAAACGCTTTTAGTTCAGAAAGTC
	**RPAP3-Cter mut2** (F630A-S632A) (IP SILAC)
Oligonucleotide forward	GTTTGATATGGCAGTGATG GCT ATG GCA GAAACAGAGAAAAAG
Oligonucleotide reverse	CTTTTTCTCTGTTTCTGCCATAGCCATCACTGCCATATCAAAC
	**PIH1D2** (2H screen)
Oligonucleotide forward	TGGAATTCGGGGCCGGACGGGCCTGGAGACATCCTCAAAAGGTCTGC
Oligonucleotide reverse	AGGTCGAGGGGCCCCAGTGGCCTCACACCAAAGGCATTGTGATGATTAGC
	**RPAP3-Cter mutants** (K584A-D589A) (2H screen)
Oligonucleotide forward	GTTGTTTCAG GCA AATCTG GCT CCA GCT GTATTCAACCAGATCG
Oligonucleotide reverse	CGATCTGGTTGAATACAGCTGGAGCCAGATTTGCCTGAAACAAC
	**RPAP3-Cter mutants** (E604A-K605A) (2H screen)
Oligonucleotide forward	GACTTTTACATT GCG GCA GAA GCG CCATTACTCATCTTTG
Oligonucleotide reverse	CAAAGATGAGTAATGGCGCTTCTGCCGCAATGTAAAAGTC
	**RPAP3-Cter mutants** (Q616A-R617A) (2H screen)
Oligonucleotide forward	CTCATCTTTGAAATCTTA GCA GCA CTTTCTGAACTAAAAAG
Oligonucleotide reverse	CTTTTTAGTTCAGAAAGTGCTGCTAAGATTTCAAAGATGAG
	**RPAP3-Cter mutants** (K636A-K637A) (2H screen)
Oligonucleotide forward	GTTTATGTCAGAAACAGAG GCA GCG ATTGCACGTG
Oligonucleotide reverse	CACGTGCAATCGCTGCCTCTGTTTCTGACATAAAC
	**RPAP3-Cter mutants** (E657A-E658A-K660A-K661A) (2H screen)
Oligonucleotide forward	GAAGGATAGTTCTGTCGCAGCACTCGCGGCAAGATACGGTGGTTGAGCCATCCAG
Oligonucleotide reverse	CTGGATGGCTCAACCACCGTATCTTGCCGCGAGTGCTGCGACAGAACTATCCTTC
