# Supplementary material for: The RPAP3-Cterminal domain identifies R2TP-like quaternary chaperones
Source: Nat Commun. 2018 May 29;9:2093. doi: 10.1038/s41467-018-04431-1 (PMC5974087; doi:10.1038/s41467-018-04431-1)
Supplement: Supplementary file 2 — Description of Additional Supplementary Files [file 41467_2018_4431_MOESM2_ESM.docx]

**Description of Additional Supplementary Files**

File Name: Supplementary Data 1

Description: Hit list for the partners identified in SILAC-IP experiments

File Name: Supplementary Data 2

Description: Hit list for the partners identified in 2H screens

File Name: Supplementary Data 3

Description: List of orthologs for studied factors
